# A Review on Optical Biosensors for Monitoring of Uric Acid and Blood Glucose Using Portable POCT Devices: Status, Challenges, and Future Horizons

**DOI:** 10.3390/bios15040222

**Published:** 2025-03-31

**Authors:** Kermue Vasco Jarnda, Heng Dai, Anwar Ali, Prince L. Bestman, Joanna Trafialek, Garmai Prosperity Roberts-Jarnda, Richmond Anaman, Mohamed Gbanda Kamara, Pian Wu, Ping Ding

**Affiliations:** 1 Xiangya School of Public Health, Central South University, Changsha 410078, China; vascojarnda@csu.edu.cn (K.V.J.); heng_dai@csu.edu.cn (H.D.); plbestman@gmail.com (P.L.B.); kamranohamedgbanda@gmail.com (M.G.K.); 2 Hunan Provincial Key Laboratory of Clinical Epidemiology, Changsha 410078, China; 3 Institute of Human Nutrition Sciences, Warsaw University of Life Sciences SGGW, Nowoursynowska 159 St., 02776 Warsaw, Poland; anwarali946@gmail.com (A.A.); joanna_trafialek@sggw.edu.pl (J.T.); 4 Xiangya School of Nursing, Central South University, Changsha 410078, China; robertsprosperity@gmail.com; 5 School of Metallurgy and Environment, Central South University, Changsha 410083, China; richmond_anaman@csu.edu.cn

**Keywords:** μPADs, optical biosensors, uric acid, blood glucose

## Abstract

The growing demand for real-time, non-invasive, and cost-effective health monitoring has driven significant advancements in portable point-of-care testing (POCT) devices. Among these, optical biosensors have emerged as promising tools for the detection of critical biomarkers such as uric acid (UA) and blood glucose. Different optical transduction methods, like fluorescence, surface plasmon resonance (SPR), and colorimetric approaches, are talked about, with a focus on how sensitive, specific, and portable they are. Despite considerable advancements, several challenges persist, including sensor stability, miniaturization, interference effects, and the need for calibration-free operation. This review also explores issues related to cost-effectiveness, data integration, and wireless connectivity for remote monitoring. The review further examines regulatory considerations and commercialization aspects of optical biosensors, addressing the gap between research developments and clinical implementation. Future perspectives emphasize the integration of artificial intelligence (AI) and healthcare for improved diagnostics, alongside the development of wearable and implantable biosensors for continuous monitoring. Innovative optical biosensors have the potential to change the way people manage their health by quickly and accurately measuring uric acid and glucose levels. This is especially true as the need for decentralized healthcare solutions grows. By critically evaluating existing work and exploring the limitations and opportunities in the field, this review will help guide the development of more efficient, accessible, and reliable POCT devices that can improve patient outcomes and quality of life.

## 1. Introduction

**Figure 1:**
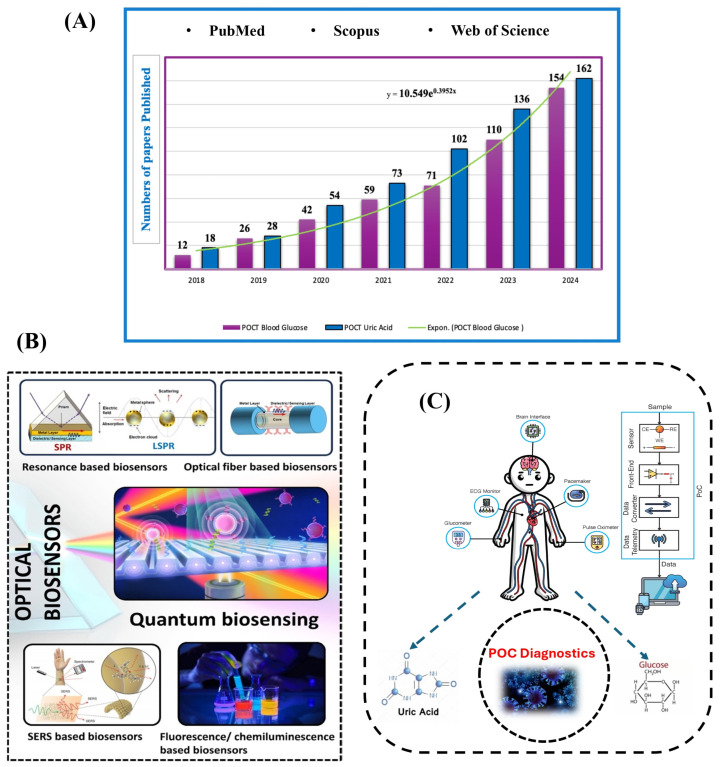
(**A**) Annual publication counts of publications utilizing the specified keywords as of 31 October 2024. (**B**) Cutting-edge optical biosensors: The figure illustrates many commonly utilized optical biosensing platforms, including resonance-based biosensors, optical fiber-based biosensors, quantum-based biosensors, and surface-enhanced Raman scattering (SERS) biosensors [55]. (**C**) Various point-of-care devices, illustrating the integration of sensors for different health-monitoring applications.

Rapid diagnostic technology development in recent years has transformed healthcare by facilitating earlier disease detection and management, which has grown in importance over time. A comprehensive analysis was performed on the Web of Science, PubMed, and Scopus databases. It has been seen that research into optical biosensors, POCT, microfluidic paper-based analytical devices μPADs, and the monitoring of glucose and uric acid has grown notably. A significant number of research articles were published on this subject between 2018 and 2024, indicating a growing interest and substantial contributions from scientists and researchers (Figure 1A).

Furthermore, other research articles have explored the topic, demonstrating the wealth of information that has developed in this field [1]. Blood glucose and uric acid levels are two of the many biomarkers used in clinical diagnostics that are essential for tracking chronic diseases, including diabetes, gout, and hyperuricemia [2,3]. Regularly checking these biomarkers is needed to keep patients healthy and avoid problems [4], but the current diagnostic methods are often still hard to use, expensive, and limited by the need for skilled staff and special tools [5,6]. Traditional approaches, which usually necessitate substantial, fixed analytical equipment, are ill-suited for regular or remote testing [7]. This has led to a greater need for diagnostic technologies that are easier to get to, cheaper, and better for people who use them. These technologies should also be able to be used outside of traditional clinical settings or at the point of care (POC) [8].

Point-of-care diagnostics offer several advantages over traditional laboratory testing, such as faster turnaround times, lower costs, and the convenience of self-monitoring, particularly for individuals with chronic conditions [9,10,11]. The potential benefit of POC devices is in enabling individuals and healthcare professionals to monitor essential health metrics in real-time, offering prompt insights for better decision-making [12]. However, accurate POC diagnostics have been hard to use in the past because of problems with portability, user-friendliness, accuracy, and how hard it is to fit many analytical functions into small devices [13]. At the moment, optical biosensors work using basic optical principles, such as absorption, fluorescence, phosphorescence, refraction, and reflection. Figure 1B displays several of these biosensors. Figure 1C also depicts various instances of POC devices along with a block diagram delineating their essential components.

Microfluidic biosensors have become a viable option for dealing with these problems, especially those that use paper as the base for manipulating fluids [14]. Adding optical detection technologies to microfluidic paper-based systems has opened up new ways to make diagnostic tools that are cheap, portable, and very sensitive [15]. μPADs offer significant advantages due to their simplicity, scalability, and ease of fabrication [16,17]. When paper is used as a base for fluidic channels, capillary-driven flow is made easier, so no extra pumps are needed, and the device is less complicated [18]. This feature, along with the ability to perform multiple analyses on a single strip [19], makes PADs perfect for point-of-care diagnostics in a wide range of situations, such as keeping an eye on biomarkers like glucose and uric acid [20,21]. Optical biosensors are essential to the efficacy of these paper-based platforms [22,23], as these tools use light signals like colorimetry, fluorescence, or surface plasmon resonance to find biomarkers at very low concentrations in a way that is non-invasive and very sensitive [24,25,26]. Optical detection is especially advantageous for point-of-care applications since it enables swift, real-time results without requiring intricate instrumentation [27]. Colorimetric assays, which rely on a visible color shift in the presence of a target analyte, can be readily observed with the naked eye or a basic smartphone camera, making them suitable for low-resource environments [28].

Uric acid, a metabolic product of purine catabolism, is pivotal in the pathophysiology of gout, a kind of arthritis impacting millions globally [29]. High uric acid concentrations in the blood, termed hyperuricemia, correlate with a heightened risk of heart disease, kidney stones, and metabolic disorder [30,31]. Traditional methods for monitoring uric acid, such as enzymatic assays [32] or high-performance liquid chromatography (HPLC) [33], are time-consuming, expensive, and not feasible for regular at-home testing [34,35]. As a result, there is an immediate need for dependable, economical, and portable sensors capable of delivering real-time readings of uric acid levels, facilitating improved disease management and prompt therapies. The examination of blood glucose monitoring is an essential component of diabetes management and has garnered much scientific attention [36,37]. Diabetes, a prevalent chronic condition worldwide, necessitates ongoing surveillance of blood glucose levels to avert both immediate and prolonged consequences [38]. Even though continuous glucose monitoring (CGM) systems are widely available, they are harder to use in places with few resources because they are expensive and require special equipment [39]. Previous investigators have utilized the devices for the concurrent measurement of uric acid and glucose, owing to their simplicity and cost-effectiveness. The progress in UA and glucose sample collection methodologies has facilitated the emergence of portable sensing devices and non-invasive testing techniques. Moreover, researchers are progressively focusing on the advancement of portable devices, or μPADs, used for field applications. These tools may include mobile phones, specialized small devices, and wearable technologies [40,41,42,43]. Some optical detection methods can be added to paper-based substrates to make these biosensors more flexible so they can find both uric acid and glucose at the same time [44,45,46]. The dual-sensing feature is particularly significant in clinical diagnostics, as the simultaneous monitoring of various biomarkers can yield a more comprehensive evaluation of a patient’s health [47]. Additionally, putting these biosensors into a portable and easy-to-use device could make these important diagnostic procedures much easier to get, and cheaper [48]. Point-of-care testing for uric acid and blood glucose monitoring is crucial for early diagnosis, real-time disease management, and personalized treatment strategies. POCT allows for the rapid on-site measurement of these biomarkers, enabling timely intervention for conditions such as gout, diabetes, and metabolic disorders [49].

Several factors affect how well these devices work, such as how long-lasting the paper substrate is [50], how sensitive and specific the optical detection methods are, and whether or not they have fluidic channels built in for accurate sample management [51]. Even though μPADs give quick results, they are often limited by things like unstable reagents, being sensitive to environmental factors, and problems with multiplexed analysis [52]. Also, the need for precise calibration and the space for human error make it hard for more people to use them, especially in places with few resources where people may not know much about technology [53,54]. This review gives an in-depth look at the present state of optical biosensors for monitoring uric acid and blood sugar at the point of care using portable microfluidic paper-based analytical devices. It also explores the technological advancements in optical biosensing, focusing on the use of colorimetric, fluorescence, and other optical detection techniques. Furthermore, it examines key challenges associated with the application of these devices in clinical settings, such as reagent stability, sensitivity, and the need for proper calibration. Finally, it discusses the future prospects of these technologies with an emphasis on improving their performance, broadening their applicability, and addressing the remaining hurdles for their widespread use in healthcare.

## 2. Biosensor

**Figure 2:**
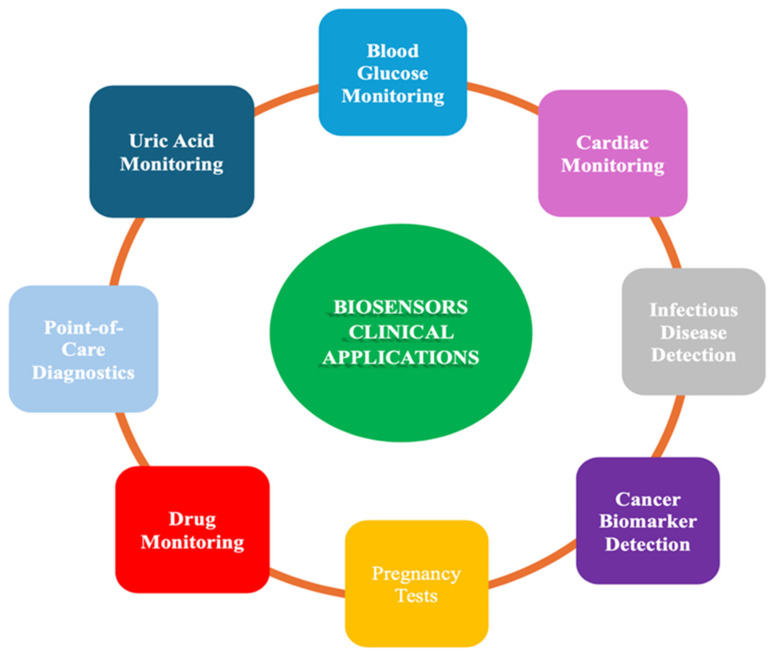
Diagrammatic representation of clinical biosensor application.

Biosensors are analytical devices, preferably of small size, that combine a biological element with a physicochemical component, generating a measurable signal aiming to detect a biological analyte [56]. They have a wide range of clinical applications, including disease diagnostics, health monitoring, and drug development, as shown in Figure 2. Biosensing platforms can use different biorecognition elements, such as antibodies, nucleic acids, enzymes and aptamers, that will bind to complementary counterparts (antigen, complementary DNA, RNA, enzyme substrate) [57]. The binding reaction causes a physicochemical change of the properties of the sample that is detected by a transducer, which converts the information into a physical quantity (electrical, optical or mass-sensitive). According to the type of transducer used, biosensing platforms can be categorized as: electrochemical biosensors, mass-based biosensors and optical biosensors [58]. To date, attention has been paid to non-invasive liquid biopsies, as they are easier to obtain, carry less risks for the patients, and generally facilitate disease monitoring.

### 2.1. Integration of Biosensors with Nanotechnology

**Figure 3:**
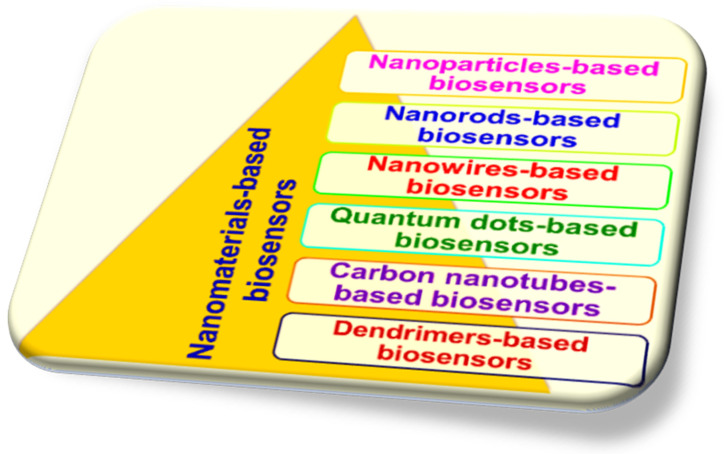
Types of nanomaterial-based biosensors [62].

The field of biosensors has undergone significant advancements in recent years due to the emergence of nanotechnology. The advancement of nanomaterial-based biosensors signifies a promising and inspiring path. These cutting-edge technologies use the special features of nanomaterials to make biological molecule detection more accurate, precise, and effective overall [59]. The incorporation of nanomaterials into biosensor design has broadened opportunities across multiple domains, including healthcare diagnostics and environmental monitoring. This integration has made it possible to quickly, accurately, and specifically identify biomolecules, which has led to new discoveries [60]. The field of biosensors is expanding significantly owing to progress in nanotechnology, research, and development. Nanomaterials like nanoparticles (NPs) made of metals and oxides, nanowires (NWs), nanorods (NRs), carbon nanotubes (CNTs), quantum dots (QDs), and nanocomposites (dendrimers) may have different properties that can be used to make biosensors work better and find things more quickly by changing their size and shape [61]. Figure 3 illustrates how different types of nanomaterials (NMs) are used to group nanobiosensors into different categories.

### 2.2. Impact and Characteristics of Biosensor as Biomarker Detector

Biosensors have emerged as powerful tools in the field of medical diagnostics and environmental monitoring, offering highly sensitive and specific methods for detecting biomarkers [63,64]. As biomarker detection becomes increasingly important for early disease diagnosis and personalized medicine, biosensors are at the forefront of this revolution. Their ability to rapidly and accurately identify molecular markers associated with various health conditions holds great promise for improving patient outcomes and advancing scientific understanding. The impact of biosensors is particularly significant in areas such as cancer detection, infectious disease monitoring, and metabolic disorders, where early intervention can greatly enhance treatment effectiveness [4].

**Figure 4:**
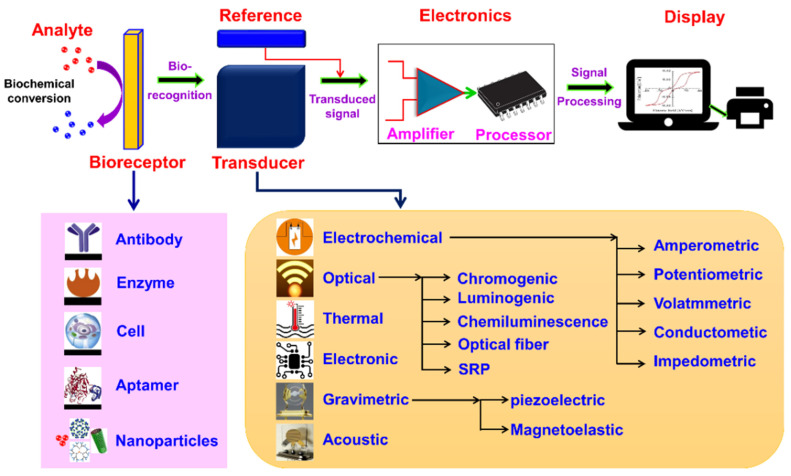
Schematic diagram of a typical biosensor consisting of the bioreceptor, transducer, electronic system (amplifier and processor), and display (PC or printer), and various types of bioreceptors and transducers used in the biosensors are also shown [62].

A biosensor is made up of four main parts: bioanalytes, a sensitive biorecognition element, a detector or transducer element, and a signal processor as shown in Figure 4 [62]. The dynamic field of biosensors demonstrates significant accomplishments through graphic representation. Frequently utilized bio-recognition elements comprise Deoxyribonucleic Acid (DNA), antibodies, tissues, enzymes, microorganisms, and cell receptors. Biosensors exhibit numerous essential characteristics such as cost-effectiveness, remarkable stability, enhanced repeatability, and increased sensitivity [56]. Table 1 discusses the use of some common biosensors in disease diagnosis. The mobility of biosensors renders them an effective instrument for detecting heavy metals. Enzyme, protein, and DNAzyme biosensors employ optical transduction to detect chemicals with enhanced sensitivity, specificity, and cost-effectiveness. This in vitro method may efficiently acquire biodegradable biomolecules with excellent selectivity [65].

### 2.3. Comparison of Point-of-Care Biosensors

**Figure 5:**
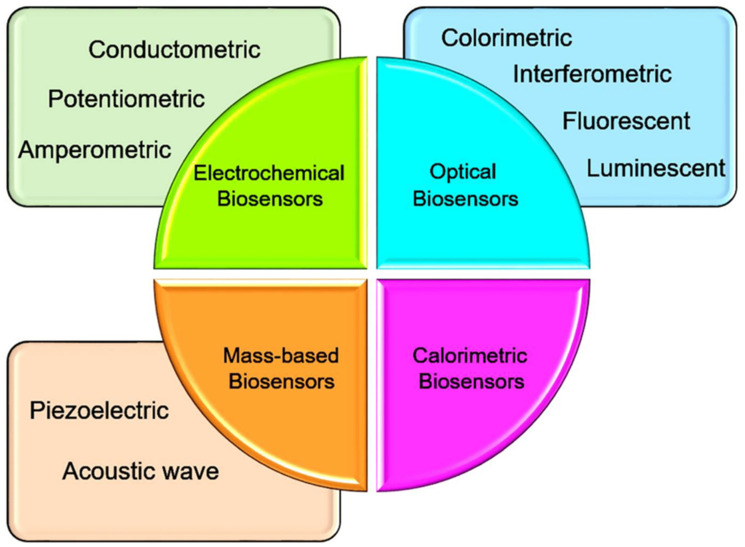
Comparative diagram of different biosensor labels and sub-classes based on the type of transducer [68].

Point-of-care biosensors vary in sensitivity, cost, and simplicity depending on their detection mechanism [66]. Electrochemical biosensors, such as glucose meters, offer high sensitivity, low cost, and simple operation, making them ideal for rapid diagnostics [67]. Optical biosensors, like surface plasmon resonance systems, provide high sensitivity but are typically more expensive and complex, limiting their portability. Lateral flow assays are highly affordable and simple to use, but may have lower sensitivity and semi-quantitative results. Microfluidic biosensors offer good sensitivity and multiplexing capabilities, but can be costly and require more technical expertise [23]. Figure 5 illustrates four different classes and sub-classes of biosensors based on the type of transducer.

**Table 1:** Use of common biosensors in disease diagnosis.

References	Analytes	Disease Diagnosis or Medical Application	Benefits	Challenges
[69]	Hydrogel-based biosensor	Regenerative medicine	Versatility, retention of bioactivity, ease of modification, water content.	Swelling and dehydration, diffusion imitations, limited mechanical strength.
[70]	Nanomaterials-based biosensor	For therapeutic applications	Enhanced sensitivity, rapid response time, multiplexed detection.	Toxicity concerns, standardization challenges, interference in biological samples.
[71]	Silicon biosensor	Cancer biomarker development and applications	Compatibility with microfabrication techniques, electronic readout, versatility in surface functionalization, long-term stability.	Brittleness, limited transparency, cost of fabrication.
[72]	Microfabricated biosensor	Optical corrections	High throughput, reduced sample volume, integration with electronics, automation compatibility.	Limited material compatibility, limited detection range, limited spatial resolution.
[73]	Uric acid biosensor	Cardiovascular and general disease diagnosis	Clinical relevance, rapid results, ease of use, portability, quantitative measurements.	Interference from other compounds, limited dynamic range, calibration requirements, sensor stability, and specificity challenges.
[74]	Glucose oxidase electrode-based biosensor and HbA1c (glycated haemoglobin) biosensor	Diabetes	High sensitivity and specificity, compact and portable, integration with electronics.	Oxygen dependency, temperature sensitivity, calibration requirements.

### 2.4. Fundamentals of Optical Biosensors

Optical biosensors are analytical devices that rely on the interaction of biological molecules with light to detect and quantify various substances [75]. The construction of these sensors typically involves a combination of a biological recognition element and a transducer, which converts the biochemical signal into a measurable optical output [62]. The biological element specifically binds to the target analyte, triggering a change in the optical properties of the sensor, such as a shift in refractive index or changes in light intensity, which can then be monitored and analyzed. Materials such as gold or silicon are often used as substrates, where they can enhance the sensitivity of the sensor by supporting the optical signal’s interaction with the biological molecules [76].

Recent developments in optical biosensors have focused on enhancing their sensitivity, specificity, and versatility for a wide range of applications. Innovations include the use of nanomaterials like nanoparticles and nanostructures to improve light–matter interactions, leading to more sensitive detections even at low analyte concentrations [77]. Miniaturization and the integration of microfluidics have also made optical biosensors more compact, portable, and suitable for point-of-care diagnostics. Also, improvements in multiplexing methods have made it possible for these sensors to pick up more than one analyte at the same time. This is especially helpful in difficult diagnostic situations like finding disease biomarkers or keeping an eye on the environment. These changes have made it possible for optical biosensors to be used in more areas, like biotechnology, medical diagnostics, environmental monitoring, food safety, and monitoring the health of the environment. They make solutions faster, more effective, and less expensive [78].

### 2.5. Classification of Optical Biosensors Based on Transduction Mechanisms

**Figure 6:**
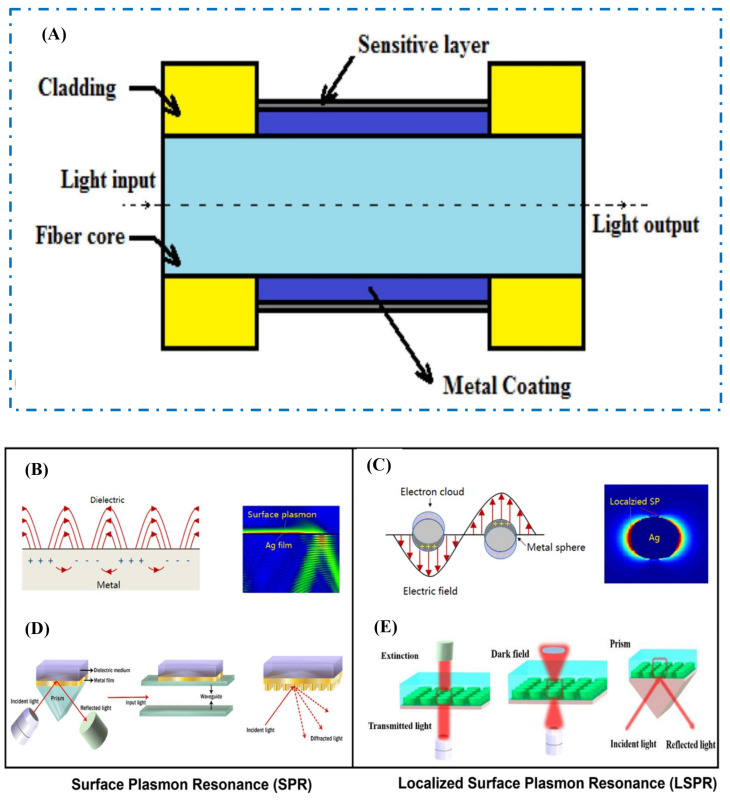
(**A**) A typical optical fiber biosensor [23]. (**B**) The generation of localized surface plasmon resonance in metallic nanostructures along with the simulation outcomes [88]. (**C**) The excitation of surface plasmon resonance by different light coupling methods for SPR biosensing [88]. (**D**) Localized surface plasmon resonance [89]. (**E**) LSPR biosensing methods, including extinction, dark-field, and prism coupler on the plasmonic nanostructured surface [89].

Researchers have committed efforts to optical biosensing, yielding diverse combinations. Figure 6 illustrates the main categories of optical biosensors, arranged according to their transduction methods. There are four primary categories of optical biosensors utilizing optical waveguides: fiber optics, surface plasmon resonance, Raman, and FTIR. Clinical diagnostics, drug development, food processing control, and environmental monitoring have all utilized optical biosensors [79].

#### 2.5.1. Fiber Optics

Fiber-based biosensors use optical fibers, which are thin, flexible strands made of high-quality glass or plastic, to find biological or chemical substances very accurately and sensitively [80]. Figure 6A illustrates how these biosensors function, utilizing analytes to interact with light passing through the fiber. Recognition components such as antibodies or DNA often alter the fiber’s surface. To find out the concentration of the analyte, changes in the evanescent wave caused by binding events are found and measured [81].

#### 2.5.2. Surface Plasmon Resonance-Optics

Surface Plasmon Resonance is an optical analytical method that has become popular in the last ten years as a useful and accurate way to find out how biological molecules interact with each other [82]. Medical analysis has dedicated tremendous efforts to the development of SPR sensing, as illustrated in Figure 6B [83]. Two types of resonance-based biosensors are the localized surface plasmon resonance (LSPR) and surface plasmon resonance sensors. These use resonance phenomena to quickly and accurately find certain chemicals or biological molecules [84]. SPR sensors quantify refractive index variations on a thin gold film resulting from target molecule binding, which is essential for biotechnology and pharmaceutical research. When light interacts with LSPR-based optical biosensors, electrons on the surfaces of metallic nanoparticles move back and forth (Figure 6C). SPR biosensors are used a lot in areas like medical diagnosis, bioimaging, food safety, antigen/antibody detection, and environmental analysis [85,86,87].

#### 2.5.3. Surface-Enhanced Raman Scattering

**Figure 7:**
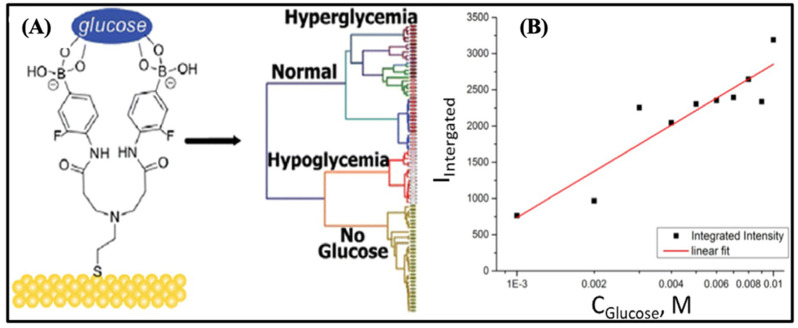
(**A**) Glucose binding with bisboronic acid functionalized the Au surface and helped in distinguishing from hypoglycemia. (**B**) Log-scale glucose concentration versus integrated SERS intensity from the concentration-dependent SERS difference spectra [92].

Surface-enhanced Raman scattering (SERS) detection is a promising method for clinical analysis because it is very sensitive, does not cost much, and also does not require much sample preparation [90]. Kong and his colleagues used a triosmium carbonyl cluster-boronic acid (Os-BA) conjugate in a study to make a new glucose measuring tool. For SERS glucose detection, a sandwich assay and a metal carbonyl probe were used [91]. This allowed for accurate glucose detection in the 1–10 mM range and the separation of spectra into hypoglycemic, normal, and hyperglycemic ranges (Figure 7A). This is the first time that a direct glucose Raman signal was used to find glucose (Figure 7B). The Raman peak intensity of 1070 cm^−3^ changed depending on the glucose concentration.

### 2.6. New Developments in Optical Biosensors

Recent advancements in materials science, nanotechnology, and signal processing are pushing the boundaries of these sensors, enabling faster, more accurate diagnostics in various fields such as healthcare, environmental monitoring, and food safety. This evolving landscape promises to revolutionize both laboratory research and point-of-care testing, offering a glimpse into a future where real-time, non-invasive detection is the norm. In a study conducted by Zhang M. et al., they reviewed fluorescence and chemiluminescence-based optical biosensors, emphasizing their use in point-of-care diagnostics. These technologies offer advantages such as high sensitivity, rapid detection times, and the potential for integration into handheld devices. The article also discusses the recent developments in molecular probes and biofunctionalization techniques, which enhance the specificity and versatility of these biosensors [93]. Cheng and his associates suggested a new dual-parameter optical fiber biosensor based on surface plasmon resonance for the simultaneous monitoring of urea and uric acid concentrations. Based on the idea of positive and negative electric combination, ZnO nanoparticles were chosen as the matrix for immobilizing urease and uricase, with the ability to selectively recognize them in their research. They can also be used as a sensitizing material to increase the refractive index detection sensitivity of SPR by 22%. The urea sensing area was then covered with a Nafion ion exchange membrane to stop the crosstalk that could happen when nearby sensing areas touched each other. Serum samples were used to validate the sensor’s ability to detect real biological samples, demonstrating the sensor’s potential for real-world applications and dependable selectivity [94]. Another study by Esposito F. et al. reported on a fiber optic biosensor for the label-free detection of vitamin D by focusing on 25-hydroxyvitamin D_3_ (25(OH)D_3_), the main form of D_3_ that circulates in the human body. The sensing platform is made up of a long period grating (LPG) that is embedded in a double cladding fiber (DCF) with a W-type refractive index profile. The device’s operating point is set to the mode transition area. Chemically etching the outside coating makes it more sensitive to the medium around it. This is because the DCF structure is made of silicon, which makes it stable over time, better at showing the grating resonance band (>10 dB), and more sensitive (up to −1400 nm/RIU). In order to add carboxylic groups (-COOH) for attaching the vitamin D3 recognition element, which is an antibody specific to 25(OH)D3, the LPG transducer is also covered with a thin layer of graphene oxide. Lastly, the biosensor’s performance was assessed in a complex media including physiological concentrations of interfering proteins, yielding encouraging findings.

**Figure 8:**
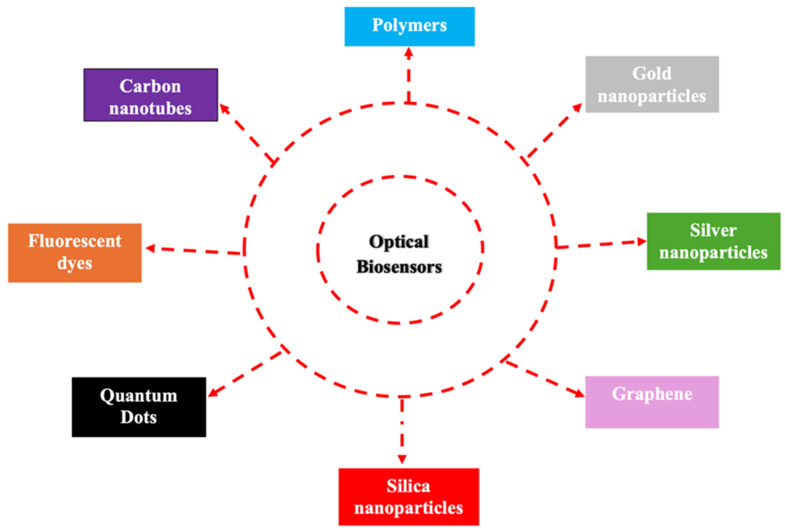
Different types of materials utilized in optical sensors on the basis of the underlying phenomenon.

Optical sensors generally rely on materials that can interact with light to detect specific changes or stimuli, such as the presence of glucose or uric acid. The most commonly used materials for optical sensors are illustrated in Figure 8. Moreover, both uric acid and glucose optical sensors are becoming more miniaturized, real-time-capable, and non-invasive through the integration of nanotechnology and advanced materials [95]. They are increasingly used in wearable health-monitoring systems, offering a less invasive alternative to traditional methods like blood draws [96]. Scalability remains a major hurdle in commercializing advanced optical biosensors for POCT due to their high production costs and manufacturing complexities. While nanomaterial-based enhancements improve sensitivity, their large-scale fabrication is costly and challenging [97]. To address this, cost-effective production methods such as roll-to-roll printing, inkjet fabrication, and semiconductor-based manufacturing can improve efficiency and reduce costs. Modular biosensor designs and open-source hardware could further enable decentralized production, making these technologies more accessible [98]. Strategic collaborations between research institutions, biotech firms, and healthcare providers are essential to achieving economies of scale, ensuring that optical biosensors transition from niche innovations to widely available, affordable diagnostic tools.

## 3. Microfluidic Paper-Based Analytical Strips (μPADs)

**Figure 9:**
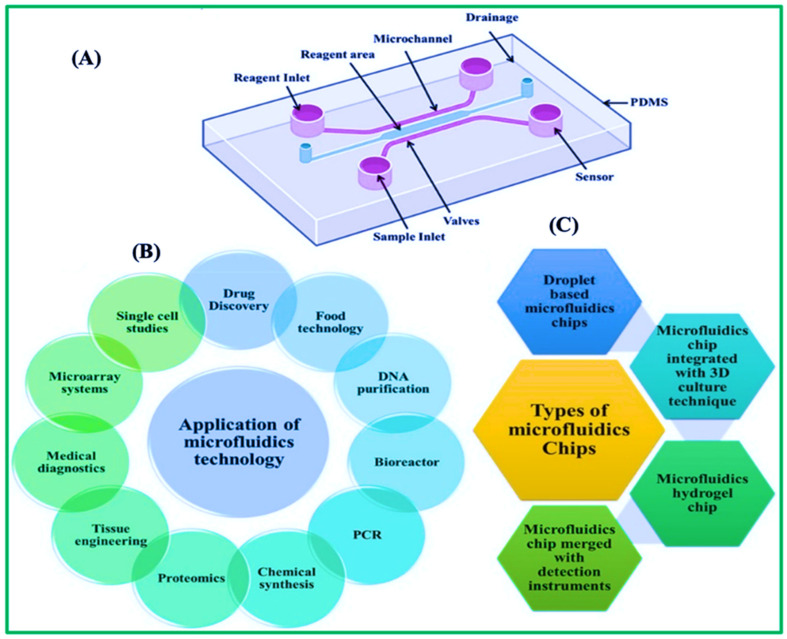
(**A**) Graphic representation of the microfluidic chips. (**B**) Types of microfluidic chips and (**C**) applications of microfluidic chips [105].

Microfluidic paper-based analytical devices have attracted considerable interest recently due to their potential use in affordable, on-site diagnostic testing [99]. In Figure 9A, these devices employ the capillary action of paper to convey fluids and carry out many biochemical experiments [18], making them well-suited for settings with little equipment [100,101]. An essential use of µPADs is detecting uric acid, a crucial biomarker for medical conditions including gout and kidney stones. Uric acid levels in the body can indicate various health issues, and it is critical to monitor these levels for effective illness management [102,103]. Recent advancements in µPAD technology have focused on improving the sensitivity, accuracy, and user-friendliness of uric acid detection, as illustrated in Figure 9B. Figure 9C illustrates the fundamental design of microchips, encompassing their components, diverse varieties, and uses [104,105]. Researchers have created innovative techniques to manufacture µPADs with greater fluidic control [106], enabling accurate sample manipulation and enhanced detection limits [107]. Microfluidics can integrate many sample processing procedures into a single chip, including extraction, labeling, and purification [108].

**Figure 10:**
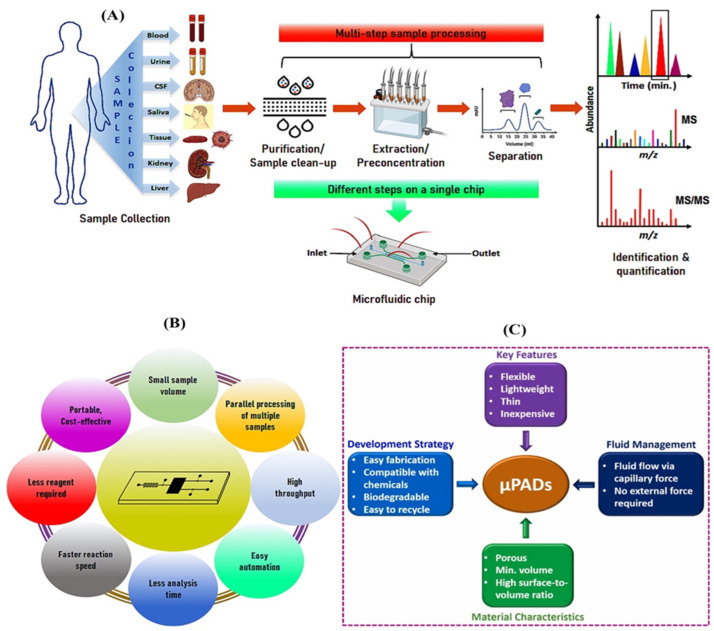
(**A**) Role of microfluidics in the general workflow of biomarker quantitation [114]. (**B**) Advantages of microfluidic chips in the field of biomarker quantitation [114]. (**C**) Key features of microfluidics-based paper analytical devices [115].

In addition, researchers have investigated the utilization of novel substances and detection methods to improve the efficiency of µPADs in detecting uric acid and glucose [109,110,111]. In addition, scientists have investigated utilizing novel substances and detection methods to improve the efficiency of µPADs in detecting uric acid [112,113]. Figure 10A illustrates the use of microfluidics in the overall process of measuring biomarker levels. Table 2 discuses some common materials that can be used in microfluidic devices. Transitioning from macroscale to tiny technologies offers advantages such as reduced sample consumption, increased analysis speed, and enhanced throughput. Selectivity and reproducibility performance are improved by integrating and automating several sample extraction processes and analytical methods. Furthermore, this method enables 1000-fold pre-concentration, as shown in Figure 10B. Figure 10C also shows the main features of microfluidics-based paper analytical devices.

### 3.1. Practical Applications and Fabrication Methods for μPADs

**Figure 11:**
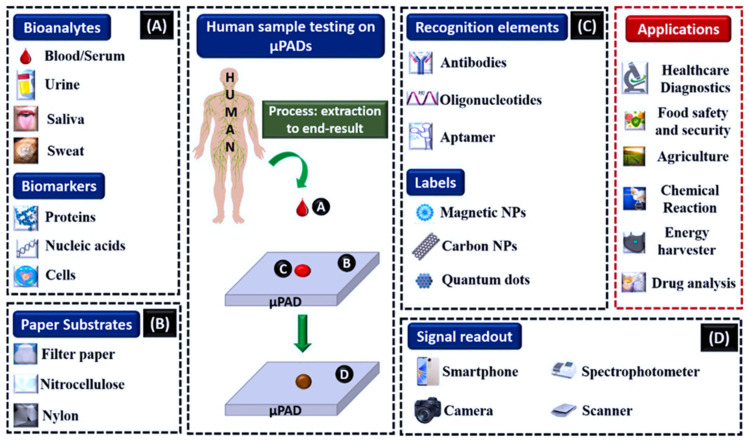
An overview of the µPAD process and its applications. Usually, µPAD-based biosensors are influenced by several development factors from (**A**–**D**) when used in numerous applications in healthcare, food, agriculture, and energy harvesting [115].

Advancements in µPAD devices incorporating modern technology have resulted in the utilization of many point-of-care testing applications. These applications include healthcare diagnostics, agriculture, environmental monitoring, energy harvesting, biochemical reactions, medicine delivery, and food monitoring [128]. Although there have been other essential investigations in this field, the current approach involves utilizing microfluidic paper-based analytical devices on a POCT platform [129]. Figure 11A–D comprehensively depicts the µPAD method and its various uses. Several development parameters influence µPAD-based biosensors. These parameters include bioanalytes and biomarkers such as blood, urine, saliva, sweat, DNA, proteins, and cells (A). They also include paper substrates (B), recognition elements (C), and signal readouts (D). These biosensors have a wide range of applications in healthcare, food, agriculture, and energy harvesting [115].

The work initiated by Muller and Clegg in 1949, which sought to create paper devices, is widely recognized as the first to contain fluidic channels. This was achieved using a paraffin-patterned fabrication technique on a paper substrate [130]. Currently, other methods of making things use something similar to a paraffin-patterned substrate to make a transparent hydrophobic barrier, which goes over the bioelectrode that is attracted to water [123]. Recently, researchers created the free application AutoPAD specifically for building paper-based microfluidic devices [18].

In their study, Ortiz-Gómez et al. created a microfluidic paper-based analytical device (µPAD) that uses colorimetry and near-field communication to find and identify glutathione (GSH) [131]. They created the design using Adobe Illustrator software and transferred it to the laser ablation machine’s controller software 1.0. The purpose was to engrave the design onto paper using a 12 W CO_2_ laser. Ag^+^’s ability to oxidize tetra-methyl-benzidine (TMB) and produce an oxidized blue dye inspired the creation of the technique [131]. Recent years have seen a variety of evaluations of the methods involving optical biosensors and procedures to produce microfluidic paper-based analytical devices (µPADs) [107,132,133,134], as shown in Table 3.

The production of microfluidic μPADs is accomplished using many techniques, including photolithography, wax immersion, cutting, stamping, spraying, embossing, screen printing, wet etching, inkjet printing, plotting, and flexographic printing [137,138]. Based on current trends, the fabrication processes of microfluidic-based paper devices are grouped into two main categories. Below, we provide a comprehensive discussion strategy for patterning μPADs.

#### Strategies for Patterning μPADs

Numerous assessments of the strategies and processes used in the production of μPADs have been conducted in recent years [139]. The fabrication of μPADs is conducted by many techniques, including photolithography, wax dipping, cutting, stamping, spraying, embossing, screen printing, wet etching, inkjet printing, plotting, and flexographic printing [140]. In this section, this review categorizes the fabrication techniques for patterning μPADs into printable and non-printable approaches.

1. Printable fabrication methods

**Figure 12:**
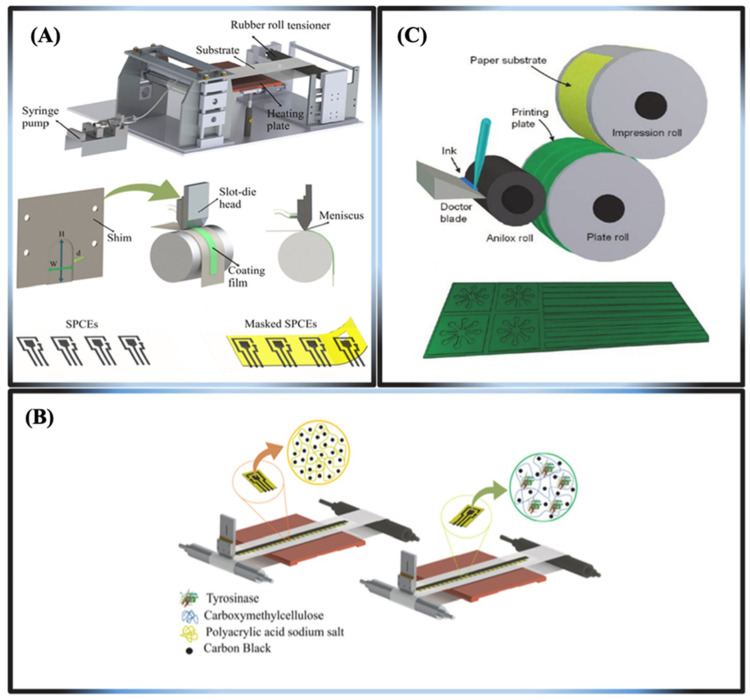
(**A**). A roll-to-roll machine is used to perform the slot-die technique through which the ink leaks through a shim and is pumped to the slot-die head [137]. (**B**) A schematic view of a fully printed electrochemical sensor with slot die coating by roll-to-roll processing for screen printing [137]. (**C**) A flexo printing unit [143].

The screen printing technique provides an efficient, economical, and rapid approach for the production of μPADs. It possesses a superior resolution compared to wax printing and an overall average resolution [141]. This approach involves creating a pattern using wax, polystyrene, and PDMS casting after constructing a screen stencil [141]. The wax screen printing technique applies solid wax to the paper through a pre-designed screen, which then melts into the paper. This method does not require a cleanroom facility, UV lamp, organic solvents, or intricate instrumentation, rendering the process convenient for the majority of users. Nonetheless, the necessity for new stencils with distinct patterns and the low resolution constrain its applicability for mass production. A notable advantage of this approach is its compatibility with the roll-to-roll process, which is widely available in many regions. Slot-die coating modifies the technique, allowing changes to the ink characteristics to adjust the film thickness (Figure 12A). This method was used to make a biosensor that can be printed out completely and uses tyrosinase to find contaminants, specifically catechol in water, as shown in Figure 12B,C [137]. Recently, there has been more interest in making 3D-μPADs using 3D printing because it is hard to put together 3D-μPADs made using other three-dimensional fabrication methods, which limits their use for mass production [142].

2. Non-printable fabrication methods

**Figure 13:**
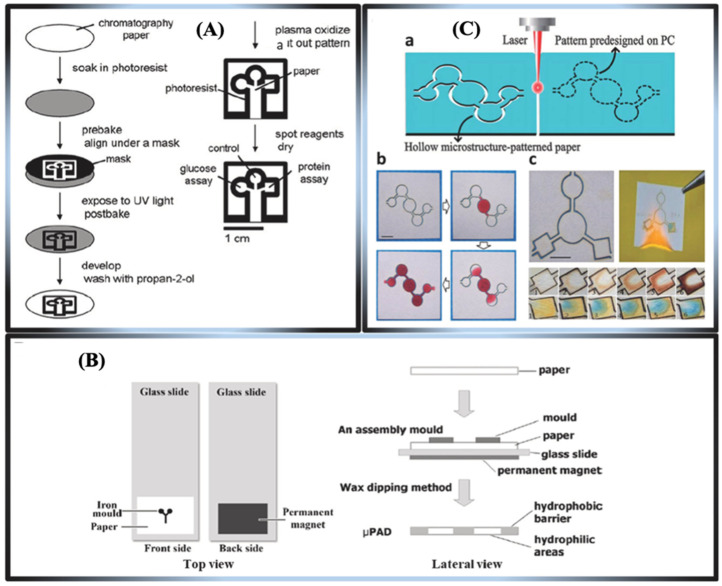
(**A**) Photolithographic method: the diagram shows the patterning method using the photolithography process, which embeds the SU-8 photoresist into the paper [115]. (**B**) Wax dipping fabrication process: The procedure for patterning paper by wax-dipping, with top and lateral view. (**C**) One-step laser cutting for creating the pattern.

In 2007, Whitesides and his team utilized photolithography as the initial patterning technique to create μPADs [115]. It facilitates the formation of a high-resolution hydrophilic–hydrophobic pattern on the paper surface. The requirement for a photomask in this method constitutes a bottleneck due to the high cost and time-intensive nature of mask fabrication. Several light-sensitive photoresist solvents, such as octadecyl trichlorosilane (OTS), poly (o-nitro benzyl methacrylate) (PoNBMA), and SU-8, can typically be used to produce the designs on masks, albeit at a relatively high cost. Furthermore, these solvents are exceedingly abrasive and adversely impact the paper’s elasticity, as illustrated in Figure 13A–C.

### 3.2. Color Spaces

**Figure 14:**
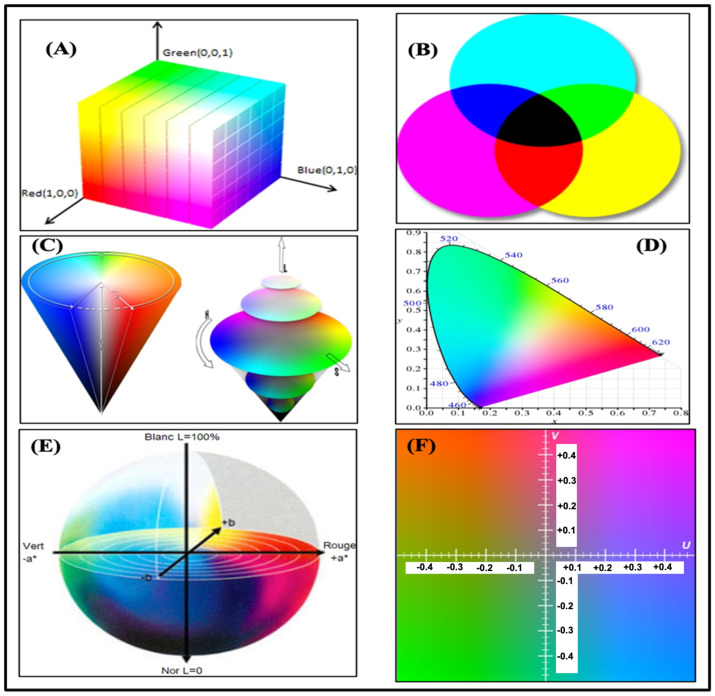
(**A**) RGB color space [144]. (**B**) CMY/CMYK color space [145]. (**C**) HSV (**left**) and HSL (**right**) color spaces [146]. (**D**) CIE XYZ color space [145]. (**E**) L*a*b* color space [145]. (**F**) YUV color space [145].

A color space, or gamut, is a collection of colors perceivable by humans or equipment. A screen or video monitor’s gamut is all the colors it can show. Figure 14A (RGB), Figure 14B (CMYK), Figure 14C (HSV/HSL), Figure 14D (CIE XYZ), Figure 14E (L*a*b*), and Figure 14F (YUV) show examples of the gamuts of different screens and monitors. Table 4 compares optical color spaces.

### 3.3. Challenges of Microfluidic Extraction Systems

Microfluidic systems have various benefits and characteristics, although they also pose obstacles and constraints. The LLE method is excellent for small-scale liquid–liquid extraction (LLE) because it can be directly connected to analytical instruments, removes contaminants well, and allows accurate extraction [151]. Nonetheless, it possesses constraints in maintaining flow stability. A segmented flow system is proposed to resolve this issue, since it provides an increased interfacial area and necessitates significant volumetric throughput. Numbering-up and multi-stream methodologies are alternate strategies. Microfluidic droplet-based liquid-phase microextraction (LPME) integrates droplet methodologies with microfluidic technologies [152]. It has better mass transfer, less adsorption, higher enrichment factors, a faster analysis time, less sample and organic solvent use, and the ability to detect things online. The organic extraction phase is less likely to be unstable when using membrane-based liquid-phase extraction methods. This is because the pores in the membrane allow for consistent laminar flow through all of the phases. Solid-phase extraction devices serve as filters, inhibiting the contamination or obstruction of equipment. Despite this, these systems involve a lot of washing steps and a difficult process, and require organic solvents for elution, which could make it so that different batches are not uniform [153]. Incorporating solid sorbents into microfluidic devices may elevate the system’s backpressure, as discussed in Table 5 below.

## 4. Biosensors for Uric Acid Detection

Uric acid is a key biomarker for several metabolic disorders, including gout, kidney disease, and cardiovascular conditions. Optical biosensors, in particular, have gained attention for uric acid detection due to their ability to provide real-time, label-free analysis with enhanced sensitivity [128]. Advances in nanotechnology have further improved the performance of optical biosensors platforms that can detect uric acid at low concentrations, paving the way for non-invasive and portable diagnostic solutions [17]. µPADs are useful for more than just finding things; they can also measure UA levels, which is important for keeping an eye on long-term conditions [164]. These devices have rapid response times, frequently within minutes, facilitating immediate medical intervention. Furthermore, by modifying the paper’s surface chemistry or adding specific biological reagents, one can easily customize µPADs for different analytes or disease markers [165]. Because they know how to use a lot of different types of detection methods, like colorimetric sensors, they are very useful in clinical settings. Researchers anticipate improvements in these capabilities with the progression of research into microfluidic paper-based analytical device technology. These changes could improve the accuracy of diagnoses and increase the number of clinical uses for µPADs in the context of finding analytes in urine [166].

### 4.1. Optical Biosensing Techniques for Uric Acid on μPADs

Traditionally, UA detection has relied heavily on laboratory environments and cumbersome, complex equipment. Recent improvements have aimed to resolve this issue by exploring portable and on-site detection devices. The aim is to enable UA analysis outside of medical environments, within the community, and in private houses. In this context, smartphones have garnered significant attention. Advancements in camera technology and the increasing sophistication of smartphones have enabled them to capture high-resolution photographs and directly process and analyze the acquired data [167]. Additionally, a few studies have utilized cloud-based servers for long-term data retention, and enabled easy access to detection results using smartphones’ networking capabilities. This has enabled the rise of telemedicine and online diagnostics.

### 4.2. Design and Fabrication Process of Optical Uric Acid μPADs

**Figure 15:**
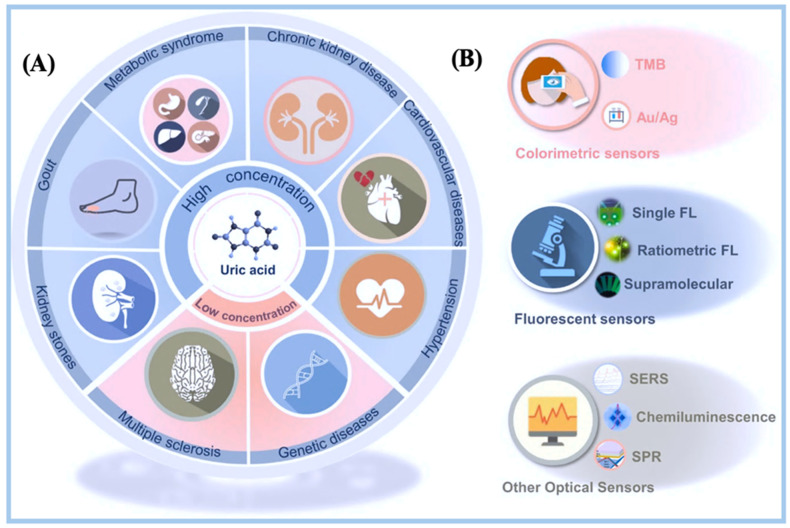
The summary of UA-related diseases and the classification of optical sensors for UA detection. (**A**) Diseases caused by abnormal UA concentration. (**B**) The classification of optical sensors for UA detection [95].

Optical UA μPADs are essential for accurate and dependable assessments of uric acid concentrations. Setting up the μPAD architecture to include fluidic channels, reaction zones, and detection regions on a cellulose substrate is part of the process. Optical detection technologies are chosen based on how sensitive they are and how well they work with the μPAD (Figure 15A), making it easier to perform both visual and quantitative evaluations (Figure 15B). The paper substrate is prepared, and printing procedures guarantee uniform dispersion and durability. Table 6 highlights recent fabrications of uric acid PADs. The incorporation of optical components, including light sources and detectors, enhances the performance of μPADs. As part of the validation, standard solutions are used to set up a reliable relationship between signal intensity and uric acid concentration. This makes sure that diagnostic applications are accurate and consistent.

### 4.3. Clinical Applications of Microfluidic Paper-Based Analytical Devices for Uric Acid Detection

In clinical settings, μPADs can be employed for the rapid on-site monitoring of uric acid levels in bodily fluids, such as urine, which is crucial for managing conditions like kidney diseases [184]. Their ability to deliver quick and reliable results makes them an attractive alternative to conventional diagnostic methods, which often require complex instrumentation and skilled personnel [185]. The application of μPADs for uric acid detection relies on the integration of various functional materials, such as enzymes, pH indicators, or colorimetric agents, onto the paper substrate [128]. These materials undergo a detectable chemical reaction in the presence of uric acid, producing a visible signal that correlates with the concentration of the analyte. This lets biomarkers in urine samples be accurately controlled and found. They are also designed to detect substances like proteins, as well as specific pathogens like bacteria or viruses, making them useful in limited-resource or point-of-care diagnostic situations [17]. µPADs can also quantify urinary tract infection levels, which are crucial for monitoring chronic illnesses like diabetes or renal problems [165]. Users can easily tailor them for various analytes or disease indicators, and this makes them highly adaptable in clinical settings [166].

### 4.4. Mobile Device Technology

**Figure 16:**
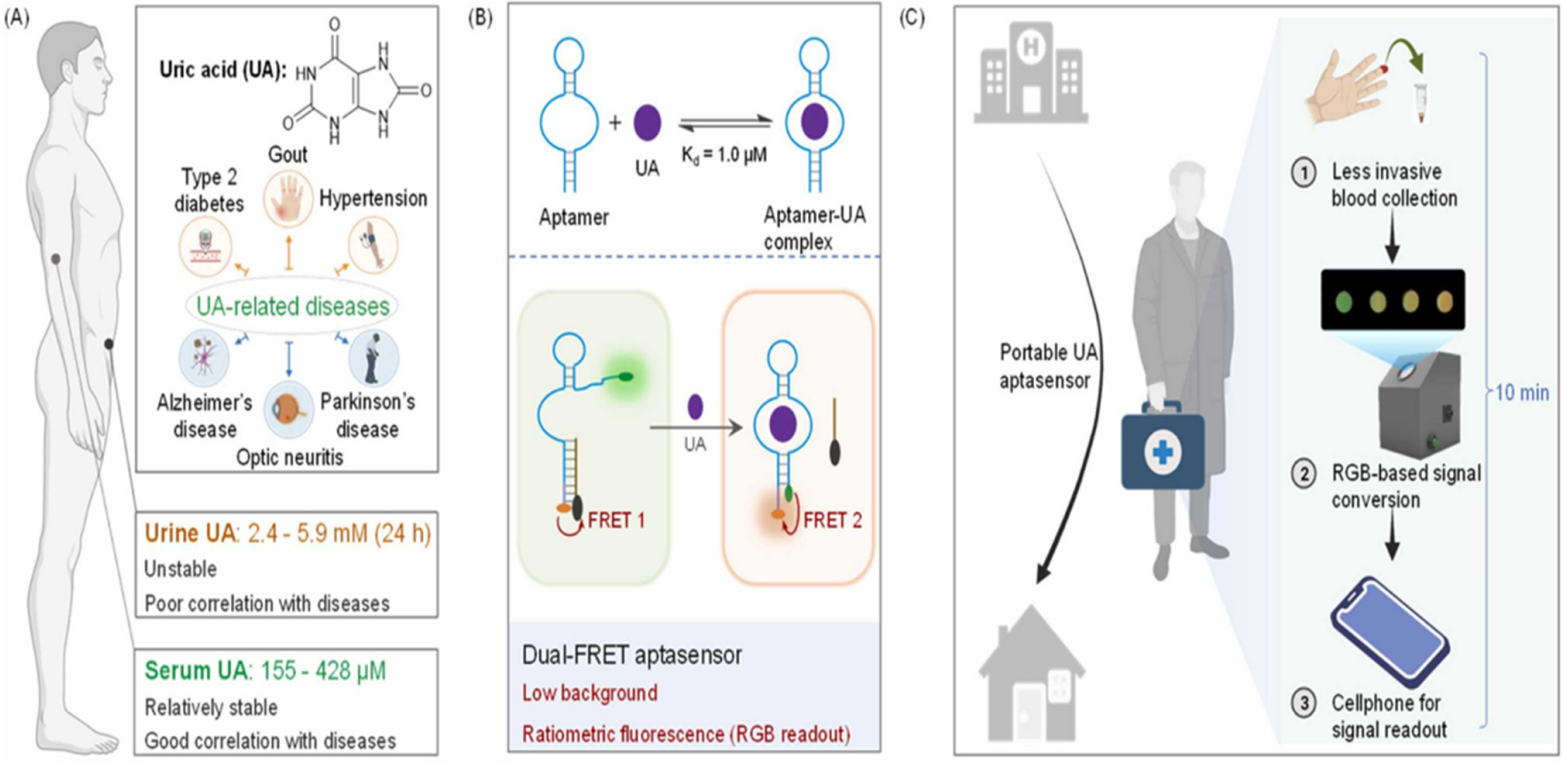
The correlation of serum UA with multiple diseases (**A**); dual-FRET aptasensor for UA detection (**B**); and facilitating UA detection with a portable 3D-printed device and RGB-based cell phone App (**C**) [187].

A prominent researcher, Li, and his research team developed a dual-FRET aptasensor with minimal signal background for serum UA detection [186]. It demonstrated a strong preference for tiny compounds that had comparable redox potentials or structures, as well as for the primary components found in serum, as shown in Figure 16A–C. The results obtained from the proposed aptasensor for analyzing clinical serum samples show a high level of concordance with those obtained from the hospital assay. 

**Figure 17:**
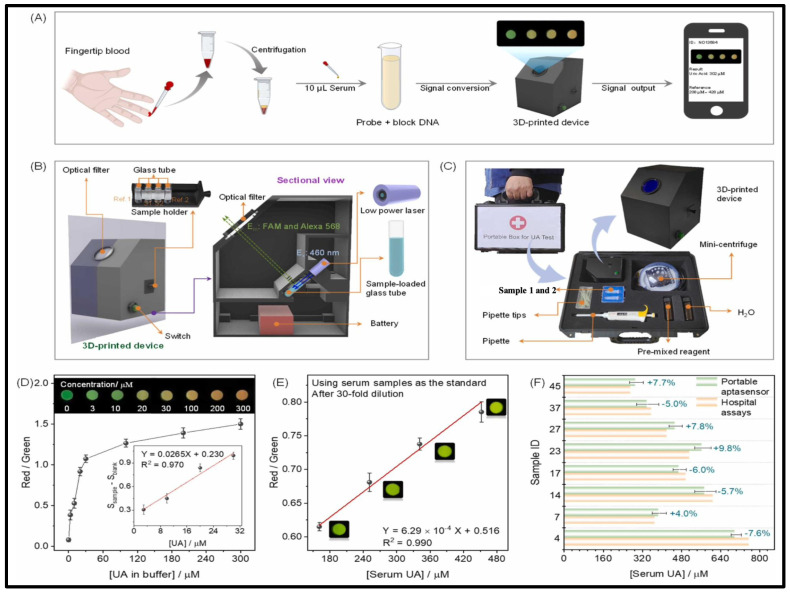
(**A**) Sketch map for UA detection. (**B**) Structural demonstration of the main components and their locations in a 3D-printed device. (**C**) Demonstration of the portable box for UA detection. (**D**) Analysis of UA from 0 to 300 μM with a 3D-printed device and RGB-based app in the buffer. (**E**) Calibration plots of human serum samples with different concentration of UA. (**F**) Validation of the accuracy of the portable aptasensor with human serum samples [187].

For the aptasensor, Lan and his research team constructed a device using 3D printing technology that could provide customized health monitoring. Additionally, they designed a mobile phone application using RGB technology to read and interpret the signals [188]. For serum UA, the portable aptasensor demonstrated a rapid detection time of 10 min. The hospital test confirmed the compatibility of the obtained results for serum samples. Furthermore, using the portable aptasensor demonstrated that serum UA exhibited more excellent stability than urinary UA. As a result, serum UA is considered more clinically relevant for personalized diagnosis. They used a mobile phone app and a portable 3D printer to facilitate UA detection, as shown in Figure 17A–F.

Zhang et al. developed a wearable microneedle colorimetric patch that uses the TMB@H_2_O_2_@ peroxidase system. When this patch is applied to the skin, it triggers a chemical reaction that changes the skin’s color in response to UA in the interstitial fluids. A smartphone can detect and capture this blue hue shift [189]. This wearable patch makes it very easy to check uric acid levels on the spot and monitor people with hyperuricemia over time. When compared to conventional blood extraction techniques, the minimally invasive patch significantly lessens patient pain and suffering, which indicates great promise for clinical point-of-care testing.

### 4.5. Specialized Devices Used for Uric Acid Detection

**Figure 18:**
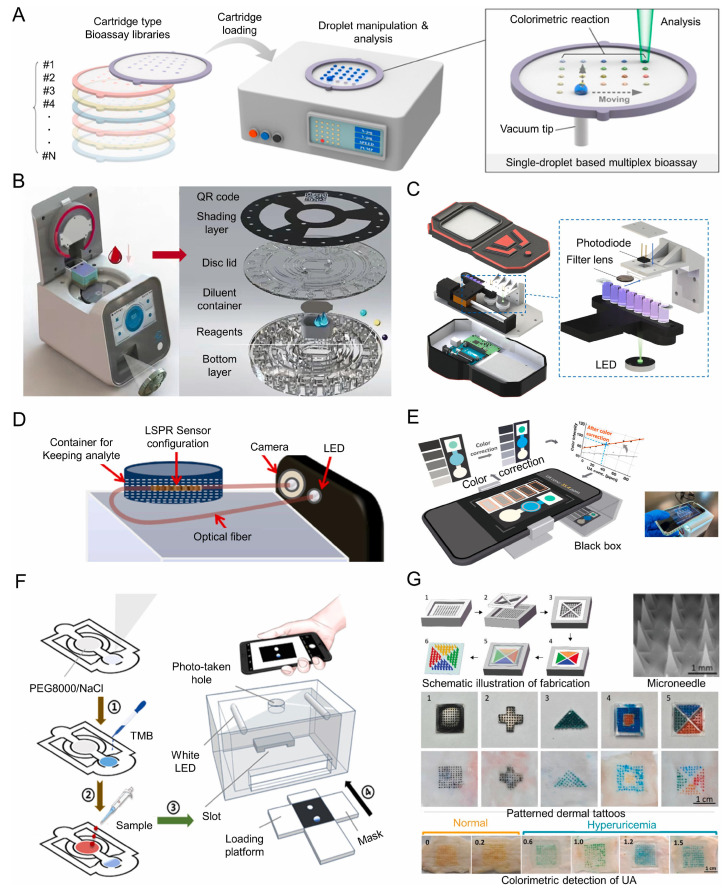
Portable devices for UA on-site detection. (**A**) Multiplex colorimetric bioassay platform based on single-droplet samples [191]. (**B**) The structural design of the centrifugal microfluidic device and its microfluidic chips [192]. (**C**) The structural design and schematic of the multi-channel handheld automatic photometer [193]. (**D**) Schematic illustration of smartphone-based fiber optic-LRSPR sensor [194]. (**E**) The schematic and the physical picture of the colorimetric analysis platform are based on paper-based microfluidic [195]. (**F**) Schematic representation of the mobile healthcare system for the detection of UA in whole blood [190]. (**G**) A schematic and physical representation of a colorimetric dermal tattoo biosensor utilizing a microneedle patch for uric acid detection [196].

The field of on-site UA detection has seen a full transformation due to the widespread usage of cell phones, which have greatly enhanced its capabilities. At the same time, potential applications for on-site UA detection have expanded due to the development of specialized equipment with enhanced precision and user-friendly operational protocols. As seen in Figure 18A–G, these discoveries show enormous promise for future development and applications in UA analysis.

Li and his colleagues developed a mobile healthcare system that operates without the need for enzymes or pre-treatment. This technology enables the on-site detection of UA through colorimetric photography. Researchers used mesoporous Prussian blue nanoparticles as nanozymes and added them to the process of inverse reduction in TMB. The researchers used a combination of TMB and H_2_O_2_ on the MPB-coated, wax-printed lithium iron phosphate (LFPs), resulting in a blue coloration. Introducing a sample drop containing UA resulted in the disappearance of the blue color, allowing detection via a smartphone rather than a UV-vis spectrophotometer. Specialized software converted RGB values from the images into greyscale values for UA calculation. The researchers developed a cloud system capable of automatically reporting UA concentration by uploading collected images [190].

Han et al. constructed a method employing various colors to identify glucose, uric acid, and lactate in small droplet samples. Super-amphiphobic polydimethylsiloxane (PDMS) acts as the substrate for the platform, whereas super-amphiphilic materials create the patterns. By applying suction to the super-amphiphobic (SPO) PDMS substrate, which is very good at repelling liquids, the vacuum tip can effectively move a droplet sample. During the traversal of SPI patterns, the droplet may release liquid droplets with volumes between 0.05 and 1.4 μL onto each SPI pattern. The droplets subsequently interact with prearranged colorimetric reagents. The surface plasmon resonance imaging (SPI) patterns of uric acid show pink marks. These marks were made when the enzyme solution containing uricase, HRP, and TRH in phosphate-buffered saline mixed with the chromogenic reagents, specifically 4-AAP and DHBS (chromogenic reagents). The SPI patterns were obtained through scanning and analyzed with Adobe Photoshop CS6 software 2.0 to ascertain the concentration of UA [191].

The results clearly show that, to some extent, the quantity of UA in body fluid that does not need to be drained can be used as a reliable indicator of serum UA levels. Therefore, when used in daily health examinations, it is equally effective in identifying, diagnosing, and tracking related illnesses. As such, the results show that it is both theoretically possible and practically important to develop wearable technology and portable on-site equipment that can be used for finding UA without touching the person.

### 4.6. Challenges in Uric Acid Monitoring from Other Analytes

**Figure 19:**
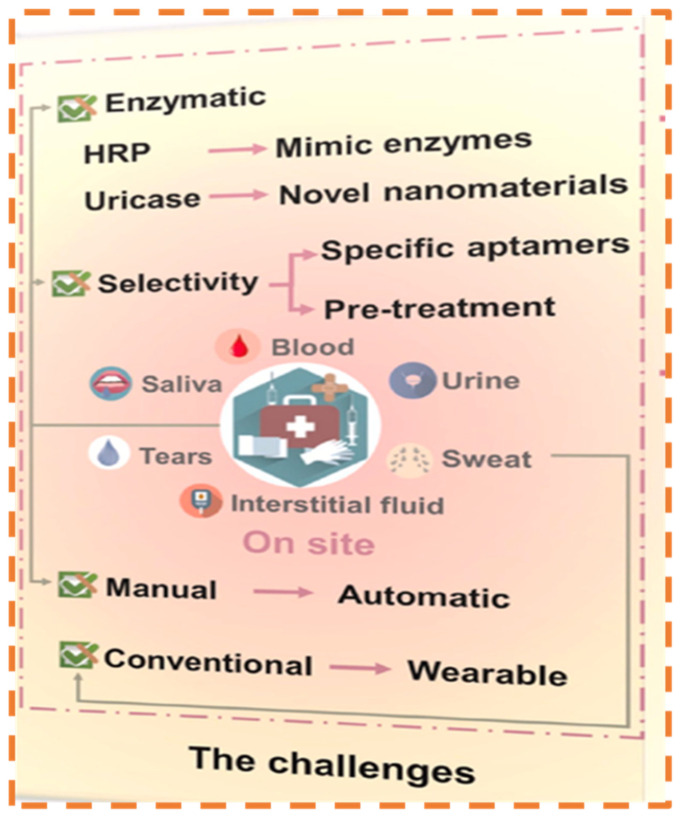
Current challenges in UA detection using optical methods [95].

The integration of optical sensors with microfluidic devices for uric acid detection remains a challenge. It is very important that different types of sensors can work together in microfluidic channels, since these platforms have to control the flow of fluids and samples, which is not always easy for optical sensors. The incorporation of optical components into microfluidic channels requires innovative design concepts and robust production techniques. Optimizing sensor performance while ensuring reliability and consistency in real-world conditions is another challenge, as shown in Figure 19. Microfluidic environments raise problems such as changing flow rates, sample matrix impacts, and biofouling, which can make sensors less sensitive and less accurate. To ensure the continuous surveillance of healthcare applications, it is critical to ensure dependable and consistent sensor operation over long periods of time. Furthermore, expanding production and guaranteeing the cost-efficiency of sensor devices combining optical and microfluidic technologies is difficult.

## 5. Biosensors for Blood Glucose Detection

### 5.1. Overview of Blood Glucose Monitoring and Its Significance

**Figure 20:**
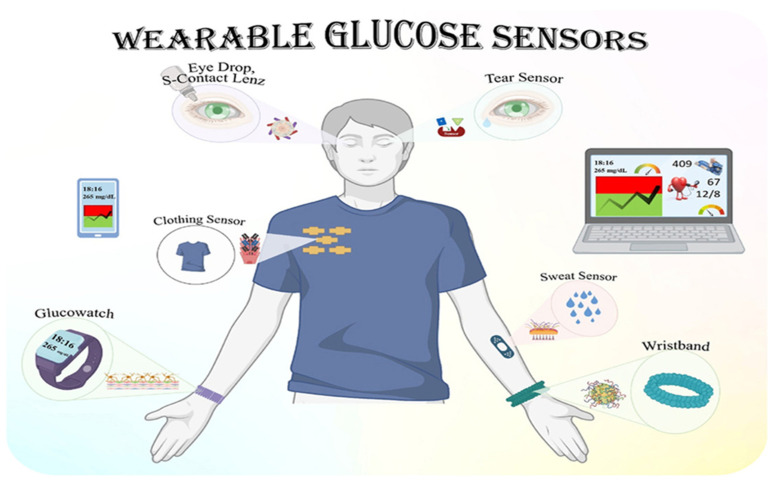
Schematic diagram of glucose biosensors used in detecting biological fluids, along with the mechanism and platform.

Blood glucose monitoring involves quantifying the concentration of glucose in the bloodstream, usually by use of a blood glucose meter. This practice is essential for patients with diabetes who must monitor and regulate their blood glucose levels. Maintaining stable blood glucose levels is crucial for preventing short-term consequences such as hypoglycemia and hyperglycemia, as well as long-term health issues including cardiovascular disease, neuropathy, and renal complications. For individuals with diabetes, consistent monitoring (Figure 20) facilitates treatment decisions, including insulin dosages and dietary modifications, to achieve optimal glucose regulation [197]. This practice is essential for identifying patterns and comprehending the influence of lifestyle factors on glucose levels. Efficient blood glucose monitoring can markedly enhance quality of life and diminish the likelihood of diabetes-related problems [198].

### 5.2. Optical Biosensing Methods for Glucose Detection on μPADs

**Figure 21:**
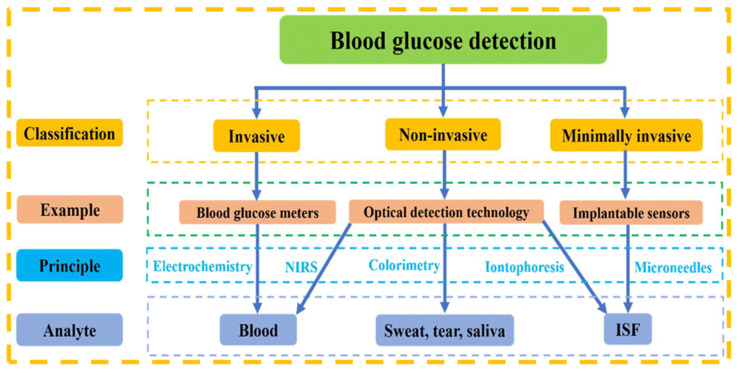
Simple classification of blood glucose detection technology [200].

These optical glucose biosensing technologies, shown in Table 7, are unique because they can be used with a variety of detection methods and give real-time results, as seen in Figure 21. Optical glucose biosensors utilize light’s interaction with living or chemical systems to convert glucose concentration into a measurable optical signal [199].

**Table 7:** Summary of glucose detection methods, nanomaterials, and limits of detection (LODs).

Analyte	Detection Method	Linear Range	LOD	Year	Ref.
Glucose	Optical	1.0–150.0 μM	0.31	2023	[201]
Glucose	Optical	20–500 μM	4.1 μM	2024	[202]
Glucose	Optical	0.5–50 μM	0.2 μM	2023	[203]
Glucose	Optical	0.2 mM to 30 mM	0.06	2023	[204]
Glucose and Hematocrit	Optical	45–630	6.4 and 9.1	2024	[205]
Glucose	Optical	2–110	5	2024	[206]
Glucose	Optical	5.0 and 20.0	25	2024	[207]

### 5.3. Recent Progress in Portable Blood Glucose Monitoring Using μPADs

**Figure 22:**
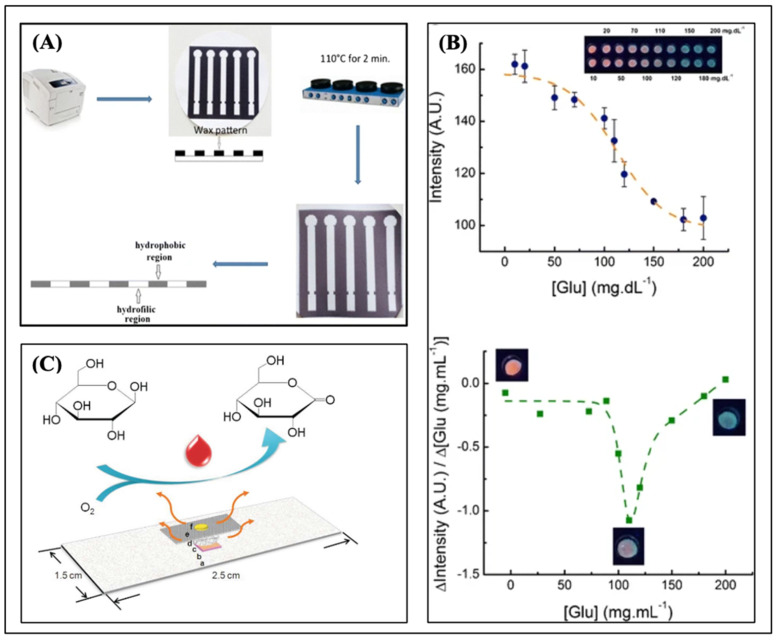
(**A**) Paper membrane-based SERS platform for the determination of glucose in blood samples [208]. (**B**) Quantum dot-modified paper-based assay for glucose screening [209]. (**C**) Turn-on paper-based phosphorescence biosensor for detection of glucose in serum [217].

The recent progress made in portable blood glucose monitoring has been huge thanks to the creation of microfluidic paper-based analytical devices (Figure 22A–C). In a recent development, Chen et al. developed a turn-on paper-based phosphorescence device utilizing Ir-Zne, a luminescent sensing material, combined with GOx through a layer-by-layer approach. Upon the introduction of glucose, the oxygen levels diminished, resulting in a concurrent increase in the phosphorescence of Ir-Zn. With a correlation coefficient of 0.9956 and a limit of detection (LOD) of 0.05 mM, the linear calibration range went from 0.05 mM to 8.0 mM [208]. Table 8 shows a comparison of several optical glucose detection systems.

**Figure 23:**
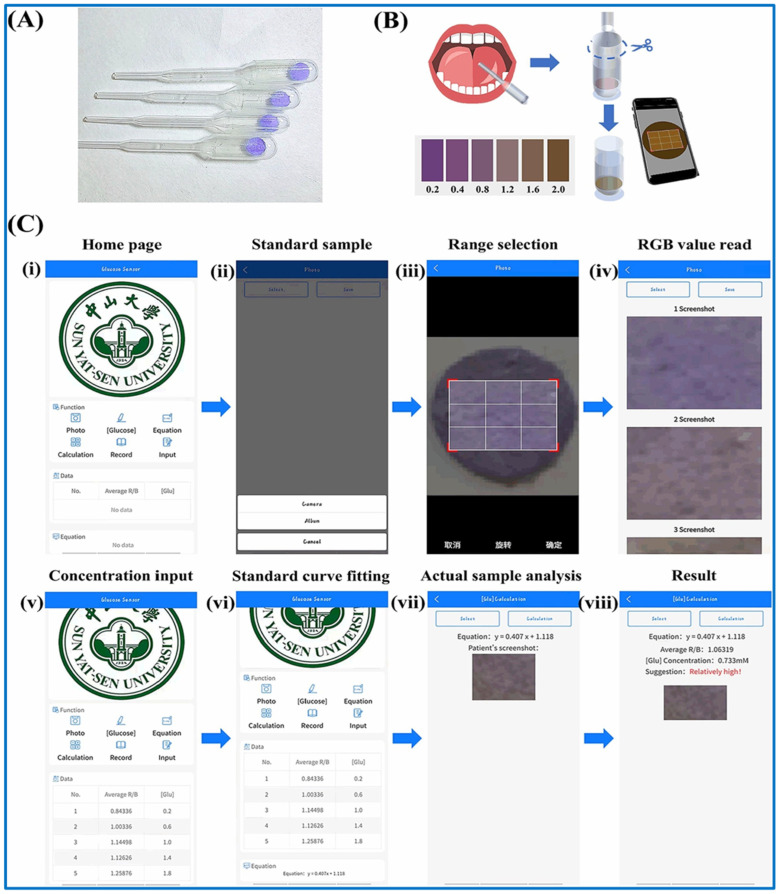
(**A**) Photo of the assembled sampling apparatus and paper chip. (**B**) Schematic illustration for the G&L@ZIF@Paper. (**C**) The operation process of the “Glucose Sensor” app used to detect glucose level [211].

Durán et al. employed colloidal CdSe/ZnS quantum dots (Q-dots) to fabricate an optical paper-based device for glucose detection. Paper infused with Q-dots would exhibit intense fluorescence when exposed to a UV lamp. Hydrogen peroxide produced by glucose oxidase may diminish fluorescence intensity following a 20 min exposure. Calorimetric detection is presented as an enhancement of existing detection methods for colorimetric μPADs [209]. A 2022 study by Hee-Jae et al. illustrated the application of a nanozyme-based colorimetric biosensor in a μPAD, facilitating quick and precise glucose detection. This method uses the catalytic properties of nanozymes to make a color change that can be seen when glucose is present. This makes readings easy without the need for complicated equipment [210]. Lin et al. also presented a smartphone-assisted “all-in-one” paper chip for the non-invasive measurement of salivary glucose concentrations, as shown in Figure 23A–C [211]. This apparatus incorporates glucose oxidase and chromogenic reagents within a metal–organic framework on a paper substrate, facilitating sensitive and user-friendly monitoring. The integration of smartphone technology facilitates quantitative analysis and real-time data exchange, improving the utility of μPADs in everyday diabetes treatment. Summarized reports of recently developed modes of portable blood glucose monitoring via optical biosensors for point-of-care diagnostics, as well as their characteristic, strengths, and limitations, are shown in Table 9. In a different study, Qian et al. showed how glucose oxidase (GOx) and the chromogenic reagent luminol can be embedded in a metal–organic framework called ZIF-67. This process is used to GOx&luminol@ZIF-67@Paper (G&L@ZIF@Paper) [211].

**Table 8:** Comparison of different optical technologies for glucose detection.

Technology	Sensing Principle	Merit	Demerit	Reference
Affinity biosensors	The competitive binding between target molecules and fluorescent	Real-time detection, miniaturization and portability, enzyme free	Sensitivity to environmental conditions, potential for non-specific binding, limited reusability	[212]
Catalytic biosensor	Glucose enzymatic reaction affects the fluorescence intensity	Fast response time, reusable, high selectivity	Complex fabrication, enzyme instability, interference from other substances	[213]
Mid-infrared spectroscopy	Specific absorption of mid-infrared photons by glucose	High molecular specificity, non-destructive analysis, broad application range	Limited penetration depth, complex sample preparation, strong water absorption	[214]
Near-infrared spectroscopy	Specific absorption of near-infrared photons by glucose	Ability to penetrate biological tissues, rapid analysis with minimal sample preparation	Overlapping spectral bands, limited sensitivity and accuracy	[215]
Raman spectroscopy	Specific vibration modes produced by the interaction of photons with glucose	Noncontact detection, less overlapped spectra, strong anti-interference	Shallow penetration depth, long acquisition time, strong fluorescence background	[92]
Polarimetry	Optical rotation of polarized light for chiral molecule	Non-destructive analysis, wide range of applications, high sensitivity to molecular structure	Limited to optically active substances, susceptibility to external factors, specialized equipment and interpretation required	[216]

**Table 9:** Summary of recently developed portable devices for blood glucose monitoring via optical biosensors for point-of-care diagnostics, as well as their characteristics, strengths, and limitations.

Infectious Disease Biomarkers	Biomarker	Transduction Mechanism	Platform	Sensor Characteristics	Pros	Cons	References
Blood glucose monitoring	Urea	Colorimetric	Smartphone	LOD: 0.19 mg/mL, LDR: 0.1–0.7 mg/mL	Minimal sample preprocessing, affordable optical phase, sensitive detection (PSD) system	Interfering substances may produce false results	[218]
	Glucose Ketone	Colorimetric	3D-printed portable photometer	LOD: 50 mg/dL and 5 mg/dL for glucose and ketone, respectively LDR: 10–1400 mg/dL and 5–160 mg/dL for glucose and ketone, respectively	Digital, frugal, portable testing device, non-invasive detection method, cost-effective	Detection of color change is negligible in low concentrations, some individuals may have ethical or legal objections to urine testing	[219]
	Glucose	Surface plasmon resonance	Optical fiber device	LOD: 3.10 mg/dL Linear range: 0–400 mg/dL	Professional operation skills are not required for operation, reusable samples can be directly loaded	Inefficient coupling of light reduces the accuracy and sensitivity, obtaining high signal-to- noise ratio is challenging	[220]
	Glucose cholesterol	Fluorescence	Paper strip	LOD:10 μmol L^−1^ for both dual-emission ratiometric fluorescent probe	Low cast, ease of operation, broad adaptability	Smartphone-based results are not reliable	[221]
	Glucose	Fluorescence	Paper strip	LOD: 2.1 μM	Excellent biocompatibility and recyclability	Vulnerable to interference and auto- fluorescence, difficulty in achieving quantitative detection	[222]
	Glucose	Colorimetric	Paper strip	Response time: 15 s LOD: 32 mg/dL Linear range: 32–516 mg/dL	Glucose monitoring via non-invasive saliva analysis, biodegradable, inexpensive test strip, fast response time	Enzymes require specific conditions to function properly, not reusable	[223]
	Glucose H_2_O_2_	Colorimetric	Paper	LOD: 10 μM for glucose and 2.5 μM for H_2_O_2_	Simple fabrication, high sensitivity, dual state (solution state and solid state) detection	Environmental factors (temp, pH, ionic strength) affect the accuracy and reproducibility of measurements	[224]

### 5.4. Continuous Monitoring of Glucose in Different Body Fluids

The ongoing surveillance of glucose concentrations in diverse bodily fluids, including blood, interstitial fluid, saliva, and sweat, represents a novel strategy for the management of diabetes and metabolic disorders [225]. Traditional finger-prick blood glucose testing is being augmented or substituted by minimally invasive and non-invasive methods employing wearable sensors and biosensors. Continuous glucose monitoring devices predominantly assess glucose in interstitial fluid, delivering real-time information that aids individuals in managing their glucose levels more efficiently [226]. Researchers are investigating alternative fluids such as saliva and perspiration to provide non-invasive monitoring techniques, while obstacles persist in guaranteeing accuracy and dependability. Improvements in nanotechnology and optical sensors are making glucose monitoring devices more accurate and useful. This makes it easier to treat diabetes and find metabolic problems early on [227], as well as comparing various qualities, including pH and viscosity, of distinct bodily fluids. The physiological concentrations of glucose in various fluids are presented in Table 10. This demonstrates that the concentration of interstitial fluids (2–22 μM) is comparable to that of glucose in blood (2–30 μM), confirming that most contemporary wearable glucose biosensors monitor glucose levels in interstitial fluids. Urine, tears, and perspiration contain glucose concentrations approximately 50 times lower than that seen in blood. In comparison to glucose levels in other bodily fluids, saliva contains the lowest concentration of glucose (0.03–0.08 μM) when measured in a healthy adult while fasting.

### 5.5. Trends of Optical Biosensors in Medical Diagnosis

Medical diagnosis is the methodical process of determining the precise disease or condition responsible for an individual’s symptoms and clinical indications. Laboratory diagnosis, a kind of medical diagnosis, frequently depends on biomarkers for identifying pathological conditions [234]. Biomarkers are measurable indications of a biological state or condition. These are the objective indicators of a medical condition that provide precise and consistent measurements [4]. Chemicals like proteins, DNA, RNA, and hormones are examples of biomarkers. They can come from cancerous tissue or other cells in the body in response to cancer or a disease [235]. Biomarkers are essential for evaluating organ function and different dimensions of health. The assessment of biomarkers typically entails the analysis of blood, urine, or soft tissue specimens. In medicine, biomarkers are critically important and indispensable in medication development. They are essential for assessing the effects of experimental medications on participants in clinical studies [4]. In addition to medical diagnoses, biomarkers can reveal exposure to or the effects of xenobiotics found in the environment and organisms [236].

## 6. Challenges and Limitations

**Figure 24:**
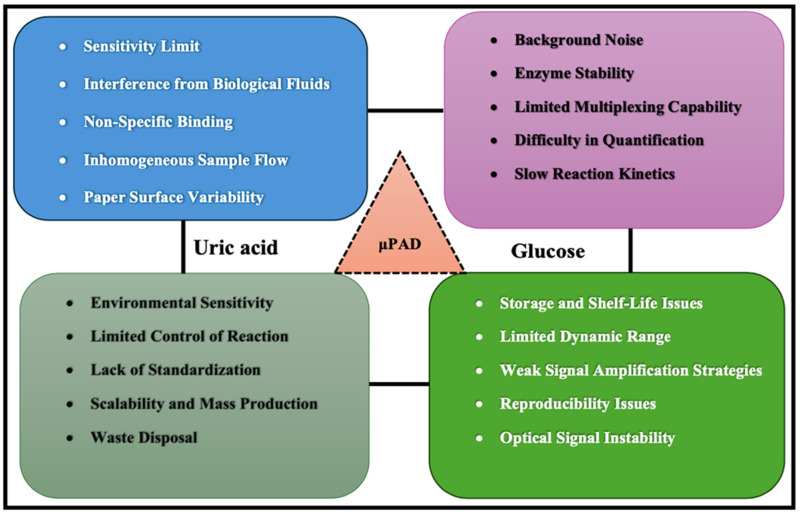
Challenges faced by microfluidic-based paper analytical devices.

Optical biosensors on microfluidic paper-based analytical devices face several challenges related to sensitivity, reproducibility, stability, and scalability [237]. Sensitivity is limited by the paper substrate’s properties, such as low optical density and uneven reagent distribution, which can cause signal attenuation and variability [238], as illustrated in Figure 24. Reproducibility suffers from manual fabrication and inconsistent paper quality, while long-term stability is compromised by biomolecule degradation and the susceptibility of paper to environmental factors like moisture and microbial contamination [239]. Scalability is also problematic, as moving from lab prototypes to mass production involves overcoming difficulties in standardizing fabrication and ensuring consistent performance across large batches [240]. The integration of advanced optical biosensors for uric acid and blood glucose monitoring into existing healthcare infrastructure presents several challenges, including data interoperability, device standardization, and clinical validation. Seamless connectivity with electronic health records (EHRs) and telemedicine platforms is crucial for real-time patient monitoring and personalized treatment. However, many current POCT devices lack standardized data formats and wireless integration, complicating their adoption in mainstream healthcare systems [241]. The biggest challenges in current point-of-care testing technologies for uric acid and blood glucose monitoring include cost, limited sensitivity, and accuracy. While many POCT devices offer rapid results, their affordability and accessibility remain issues, especially in low-resource settings [242].

Signal amplification on porous substrates and interference from other metabolites further complicate accurate measurements [243]. User-friendliness is another concern, especially for non-experts, as the sensors must have easy sample application [244], clear readouts, and simple processing steps to allow widespread adoption in home or point-of-care settings. Environmental factors like temperature and humidity can significantly impact sensor performance. Temperature variations may affect enzyme activity or biomolecule stability, while moisture can degrade the paper substrate or interfere with the reactions [245]. To overcome these issues, innovative strategies such as integrating nanomaterials to enhance sensitivity [246], improving surface modification techniques for better reagent immobilization, and developing interference-resistant sensor designs are needed. Additionally, protective coatings or encapsulation methods can help mitigate environmental effects, improving the overall reliability of optical biosensors on μPADs.

Uric acid is often used in diagnostic technologies, such as sensors for monitoring metabolic conditions; it can be used as an indicator of oxidative stress or as a marker in certain health conditions like gout. The technology might be operated through electrochemical sensors or spectroscopic methods, whereby uric acid levels are measured via interaction with specific reagents or electrodes. While measuring uric acid can help diagnose certain conditions, its specificity and accuracy could be affected by interference from other compounds in the body. The technology may also have issues with sensitivity, and the devices could require calibration or be prone to errors due to environmental factors.

Glucose monitoring, especially for diabetes management, typically relies on sensors that detect glucose levels in blood or interstitial fluid. Technologies like continuous glucose monitors (CGMs) or glucometers often use enzymes like glucose oxidase to convert glucose into hydrogen peroxide, which then generates an electrical signal correlating to glucose concentration. Despite advances in glucose monitoring, issues like sensor drift, lag time between blood glucose levels and sensor readings, and discomfort from invasive methods persist. Non-invasive options, like optical sensors, face challenges in terms of reliability and accuracy. There is also the limitation of glucose fluctuations due to factors like diet or exercise that might not always be reflected promptly.

Addressing these challenges requires a multi-disciplinary approach, including collaborations between sensor developers, regulatory bodies, and healthcare providers to establish standardized protocols and accelerate approval timelines. By tackling these integration and regulatory barriers, optical biosensors can transition from research innovations to clinically viable tools, improving accessibility and efficiency in POCT diagnostics.

### Future Horizons

Improvements in materials and design advancements are anticipated to substantially elevate the efficacy of biosensors and diagnostic instruments. The incorporation of sophisticated nanomaterials, including graphene-based structures and functionalized nanoparticles, can enhance sensitivity, selectivity, and durability [247]. Moreover, the advancement of intelligent substrates with adaptive characteristics, such as self-cleaning or stimuli-responsive functionalities, would enhance biosensing capabilities, facilitating more dependable and effective diagnostics. A promising avenue is the seamless integration of biosensors with mobile devices for real-time monitoring and data analysis [248]. The emergence of smartphone diagnostics and wearable biosensing technologies will enable continuous health monitoring, individualized treatment, and telehealth services. Utilizing cloud computing and artificial intelligence, biosensors will gather data and deliver actionable insights, equipping consumers and healthcare professionals with prompt and accurate health evaluations [249]. The capacity for the multiplexed detection of various analytes within a single assay signifies a significant advancement in diagnostics. The capacity to concurrently assess biomarkers like uric acid, glucose, and other essential indicators will transform disease monitoring and management.

Nonetheless, attaining widespread use necessitates surmounting considerable regulatory obstacles. Standardization, validation, and adherence to healthcare legislation will be essential for commercialization [250]. The future of biosensing technology is still very bright, with the potential to revolutionize early illness diagnosis and individualized healthcare as researchers and industry leaders strive to overcome these obstacles [251].

Emerging technologies in optical biosensors for the point-of-care testing of uric acid and blood glucose are being enhanced with nanomaterial-enhanced sensing, photonic crystals, and plasmonic-based detection to improve sensitivity, specificity, and miniaturization [156]. Wearable and non-invasive approaches, such as sweat- or tear-based glucose monitoring using integrated optical sensors, are gaining traction as alternatives to traditional blood-based methods. Additionally, quantum dots and SERS are underexplored yet promising solutions that could significantly enhance detection limits and multiplexing capabilities [252]. Despite these advancements, commercial adoption remains slow due to regulatory hurdles, cost constraints, and integration challenges with existing POCT platforms. Exploring AI-driven spectral analysis and lab-on-chip innovations could further bridge these gaps, leading to more efficient and accessible monitoring solutions [253].

**Figure 25:**
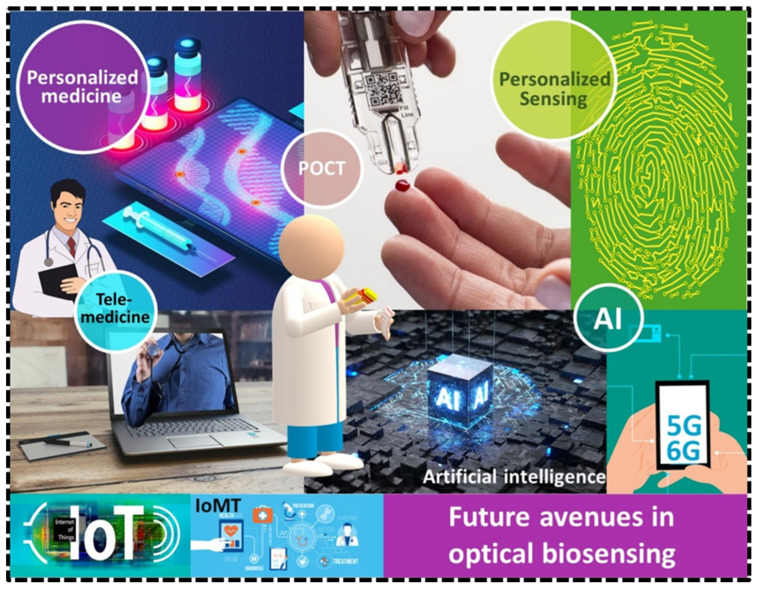
The future scope of optical biosensing—optical biosensors in future will be integrating with advanced technologies such as artificial intelligence (AI), Internet of Things (IoTs, IoMTs) and 5th/6th generation technology, which will contribute to an advanced ecosystem of personalized medicines and personalized POC sensing [55].

Moreover, integrating optical biosensors with AI-driven diagnostic tools has the potential to revolutionize POCT by enhancing both patient outcomes and data management. AI can significantly improve sensor accuracy by analyzing complex data patterns, offering real-time insights, and enabling predictive diagnostics [254]. For example, AI algorithms can process data from optical sensors to detect early signs of anomalies in uric acid or glucose levels, providing personalized treatment recommendations and improving disease management [255]. Furthermore, AI-powered data management systems can streamline the integration of sensor data into electronic health records, allowing for seamless patient monitoring and improving communication between patients and healthcare providers [256]. Future avenues in optical biosensing are depicted in Figure 25. This integration could optimize the efficiency of POCT devices, making them more responsive, scalable, and capable of offering continuous, personalized care.

## 7. Conclusions

This review highlights the potential use of point-of-care optical biosensors for uric acid and glucose in healthcare. These innovative devices offer rapid and accurate diagnostics, providing near-patient diagnosis and monitoring. They allow for personalized treatments, reduced healthcare costs, and improved diagnosis outcomes. Microfluidic paper-based analytical devices have shown great promise when used as platforms for optical biosensors to detect uric acid and glucose. This device has demonstrated considerable advances and shows enormous potential for various applications. Using microfluidics along with an optical detection method makes them cost-effective to produce, reduces the sample volume needed, and makes them portable, making them perfect for point-of-care diagnostics when resources are limited. Nevertheless, there are still obstacles to overcome, such as the requirement for improved sensitivity and repeatability, as well as the necessity to optimize stability and shelf-life for practical use in clinical and field applications. In order to ensure the future success of μPADs in uric acid and glucose detection, it is crucial to tackle these issues by making more progress in materials research, microfluidic design, and sensor integration. Integrating nanomaterials to improve sensitivity, developing biosensors with the ability to detect many analytes, and enhancing manufacturability for large-scale manufacturing are essential in expanding the usefulness of μPAD-based biosensors. Furthermore, exploring innovative manufacturing methods and integrating smartphone-based reading systems could improve the accessibility of fast and dependable uric acid testing. In the future, μPADs have the potential to enhance diagnostic capabilities and contribute to the progress of customized healthcare and biomedical research through continuous research and interdisciplinary collaborations.

## Figures and Tables

**Table 2:** Summary of some common materials used in microfluidic devices.

References	Analytes	Compatible Solvents	Fabrication Methods	Channel Dimension	Working Temperature (°C)
[116,117]	Polyimide	Most solvents	Photopolymerization; casting	<100 nm	<400
[104]	Paper	No organic, surfactant solvents	Etching, printing	~200 µM	<30–50
[118]	Thermoplastics	Good solvent compatible	Thermomoulding	~10 µM	High
[119]	Poly-methyl methacrylate (PMMA)	No alcohol, acetone, benzol	Thermomoulding, 3D printing	~100 nm	<60–100
[105]	Fluorinated ethylene propylene	Most solvents	Thermomoulding	~100 nm	<200
[120]	Hydrogel	No most solvents	Casting; photopolymerization	~10 µm	<25–32
[121]	Elastomer	Less solvent compatible	casting	<1 µm (3D)	Medium
[122]	Perfluoroalkyl (Teflon PFA)	Most solvents	Thermomoulding	~100 nm	<125
[123]	Silicon	Most solvents, no potassium hydroxide (KOH)	Etching	<100 nm	<1415
[124]	Polycarbonate	No KOH, ketones, acetone	Thermomoulding	~100 nm	<260
[125]	Polydimethylsiloxane (PDMS)	No most organic solvents	Casting	~20 nm	<40–50
[126]	SU-8 photoresist	Most solvents	Casting; photopolymerization	~100 nm	<150
[127]	Thermoset	High solvent compatible	Casting, photopolymerization	~100 nm	High

**Table 3:** Materials used in microfluidic chip fabrication for biomarker study in different body fluids.

References	Disease	Biomarker	Technique	MOC	Matrix	LOD
[135]	Periodontitis	Nitrite	Optical	μPAD	Saliva	10 μmol/L
[136]	Prognosis in chronic heart and kidney disease	Creative protein (CRP)	Optical	μPAD	Plasma and serum	54 ng/mL

**Table 4:** Comparison of color spaces for analysis of optical/colorimetric detection.

References	Color Space (Primary Parameters)	Color mixing	Merit	Demerits
[147]	Y (luminance), U (blue chroma), V (red chroma) I (rotated from U), Q (rotated from V)	Additive	Better separation of luminance and chrominance, alignment with human perception, convenient for image acquisition and display.	Non-uniform, illumination, color is not linear.
[148]	Cyan, magenta, yellow, and black	Subtractive	Precision in color mixing, better color, reproduction for printing, efficient use of ink.	Limited gamut, difficult to achieve bright color, complex color management.
[149]	L: Luminance a: red to green b: blue to yellow u: Saturation v: Hue angle	Additive	Perceptual uniformity, device-independent, better representation of human vision.	Not intuitive for users, limited gamut coverage, mathematical complexity.
[146]	Hue, saturation, value hue, saturation, intensity	Additive	Intuitive for artists and designers, better control over color adjustments, effective for color selection.	Not physically accurate, limited for complex color representation, no true perceptual uniformity.
[150]	Red, green, blue	Additive	Intuitive for displays, wide support in digital media, efficient for additive color mixing.	Not ideal for print, limited range for some colors, device dependency.

**Table 5:** Advantages and disadvantages of optical biosensors in point-of-care diagnostics: non-invasive, rapid, and real-time assessment.

References	Challenges	Advantages	Disadvantages
[154]	Non-invasive	Minimizes patient discomfort and risk of infection.	May lack the ability to detect biomarkers at very low levels.
[155]	Rapid	Provides quick results, enabling timely decision-making.	Requires precise calibration to maintain accuracy.
[156]	Real-time analysis	Allows for continuous monitoring of patient conditions.	Susceptible to environmental interference
[157]	User-friendly	Easy to use for medical personnel with minimal training.	May require expensive equipment for certain applications.
[158]	Portability	Compact and suitable for use in diverse settings.	Limited battery life or power requirements can be a constraint.
[159]	Sensitivity	High sensitivity for specific biomarkers.	Cross-reactivity may result in false positives.
[160]	Specificity	Targeted detection reduces misdiagnosis.	Narrow detection range may overlook broader health indicators.
[161]	Cost-effectiveness	Reduces overall diagnostic costs in some cases.	Initial setup costs for devices may be high.
[162]	Scalability	Easily adaptable for mass screening in public health.	May not be suitable for all biomarkers or diseases.
[163]	Environmental impact	Reduces waste by eliminating invasive consumables.	Sensor components may involve complex recycling.

**Table 6:** Recent fabrications of µPADs, such as paper-based continuous microfluidic flow (p-CMF) and paper-based DMF (p-DMF) devices, rely on UA detection.

References	Technique of Fabrication	Materials	Channel (μM)	Barriers (μM)	Advantages	Disadvantages	Devices
[142,168,169,170]	3D Printing	Conductive/non-conductive filament	541	490	Fast process; accessible to mass production.	Expensive, resolution depends on the type of printer	3D printer with a custom-made extruder
[171]	Polydimethylsiloxane printing (PDMS)	PDMS	285	1100	Cheap, easily adaptable and transparent.	Low resolution, permeability	Desktop plotter
[172]	Punching	Wax/polystyrene/conductive ink; screen stencil	210	850	Flexibility; ease of application.	Durability, environmental impact	Hot plate or oven (for heating), Commercial ink
[173]	Screen printing	Polystyrene; wax	129	321	Affordability, versatility.	Output quality, durability	Customized masks kit
[174,175]	Spraying	Mask, back supporting plate, hydrophobic coating material	532	900	Uniform coating, reduced waste.	Process complexity, quality consistency	Hand-held spray tool with customized masks
[176,177]	Wax printing	Wax	561	467 ± 33	Rapid prototyping, high resolution; suitable for mass production.	Low tenacity by use of wax, temperature sensitivity	Wax printer; hot plate
[178]	Casting	Metal substrate	245	56	Cost effective; design flexibility.	Dimensional accuracy, material limitations	Casting tools and equipment
[179]	Inkjet printing	Alkyl ketene dimer UV-curable acrylate	190	810	Low cost; energy efficiency.	Resolution limitations, quality control	Inkjet printer (conductive/non- conductive ink)
[180,181,182]	Chemical vapor deposition	Novel materials and hydrophobic chemicals, chlorosilane	624	125	High purity films, conformal coating.	Slow deposition rate, surface contamination	Vacuum chamber, heat block, hot plate
[183]	Photolithography	Photoresist and developer, photomask	186	248	High resolution, scalability.	Costly, complex fabrication procedure	Photomask, UV light source (sunlight, UV lamp)

**Table 10:** The physiological concentrations of glucose in different fluids.

Body Fluids	pH	Viscosity	Glucose Concentration Range	References
Sweat	4.5–7	0.92	0.02–0.6 μM	[228]
Tear	6.5–7.6	1.5–3	0.1–0.6 μM	[229]
Saliva	6.2–7.6	2–8	0.03–0.08 μM	[230]
Interstitial fluid	7.2–7.4	1–10	2–22.2 μM	[231]
Urine	4.5–8	0.6–1.2	0–0.8 μM	[232]
Blood	7.35–7.45	1.52–1.54	2–30 μM	[233]

## Data Availability

Data sharing is not applicable to this article as no datasets were generated or analyzed during the current study.

## Abbreviations

4-AAP and DHBS	Chromogenic Reagents
Ag TNPs	Silver Triangular Nanoprisms
AKD	Alkyl Ketene Dimer
CE	Capillary Electrophoresis
CE	Counter Electrode
CNC	Computer Numerical Controlled
CNs	Carbon Nanotubes
CO_2_	Carbon dioxide
COC	Cyclic Olefin Copolymer
DNA	Deoxyribonucleic acid
DPV	Differential Pulse Voltammetry
EIS	Electrochemical Impedance Spectroscopy
FEP	Fluorinated ethylene propylene
FI	Fluorescein
FLASH	Fast Lithographic Activation Sheets
FRET	Förster Resonans Energy Transfer
GC	Glassy Carbon
GC5A	Guanidinocalix [5] arene
GNPs	Gold Nanoparticles
GNRs	Gold Nanorods
GSH	Glutathione
H_2_O	Water
H_2_O_2_	Hydrogen Peroxide
HAuC_l4_	Chloroauric acid
HPLC-UV	High-Performance Liquid Chromatography–Ultraviolet
HPLC	High Performance Liquid Chromatography
HPRT	Hypoxanthine–Guanine Phosphoribosyl Transferase
HRP	Horseradish Peroxidase
ICP-MS	Inductively Coupled Plasma Mass Spectrometry
IDA	Indicator Displacement Assay
LLE	Liquid–Liquid Extraction
LPME	Liquid-Phase Microextraction
LSPR	Localized Surface Plasmon Resonance
mHealth	Mobile Healthcare
MIP	Molecularly Imprinted Polymer
MPBs	mesoporous Prussian blue nanoparticles
MS	Mass Spectrometry
MWCNT	Multi-Walled Carbon Nanotubes
nFCD	Non-Fluorescent Carbon Dots
NMs	Nanomaterials
NPs	Nanoparticles
NRs	Nanorods
NWs	Nanowires
O2	Oxygen
p-CMF	paper-based continuous microfluidic flow
PB	Prussian Blue
PBS	Phosphate Buffer Solution
PDMS	Polydimethylsiloxane
PFA	Perfluoroalkyl
PGMs	Personal Glucose Meters
PMMA	Poly methyl methacrylate
POCT	Point of Care Testing
PSA	Prostate-Specific Antigen
PZT	Piezoelectric Actuator
QDs	Quantum Dots
RE	Reference Electrode
RGB	Red, Green, Blue
SERS	Surface-Enhanced Raman Spectroscopy
SPE	Solid-Phase Extraction
SPI	Super-Amphiphilic
SPI	Surface Plasmon Imaging
SPO	Super-Amphiphobic
SPR	Surface Plasmon Resonance
SWV	Square Wave Voltammetry
TMB	Tetra-Methyl-Benzidine
UA	Uric Acid
WE	Working Electrode
XDH	Xanthine Dehydrogenase
μPADs	Microfluidic Paper-Based Analytical Devices
